# Anti-colon Cancer Effects of *Dendrobium officinale* Kimura & Migo Revealed by Network Pharmacology Integrated With Molecular Docking and Metabolomics Studies

**DOI:** 10.3389/fmed.2022.879986

**Published:** 2022-06-30

**Authors:** Shengchang Tao, Jinyan Li, Huan Wang, Shaobo Ding, Weichao Han, Ruirong He, Zhiyao Ren, Gang Wei

**Affiliations:** ^1^School of Pharmaceutical Sciences, Guangzhou University of Chinese Medicine, Guangzhou, China; ^2^Department of Pharmacy, Affiliated Dongguan Hospital, Southern Medical University, Dongguan, China; ^3^The Research Centre of Chinese Herbal Resource, Shaoguan Institute of Danxia Dendrobium Officinale, Shaoguan, China; ^4^Department of Systems Biomedical Sciences, School of Medicine, Jinan University, Guangzhou, China; ^5^NHC Key Laboratory of Male Reproduction and Genetics, Guangzhou, China; ^6^Department of Central Laboratory, Family Planning Research Institute of Guangdong Province, Guangzhou, China

**Keywords:** *Dendrobium officinale*, UPLC-ESI-MS/MS, network pharmacology, colorectal cancer, molecular docking

## Abstract

**Objective:**

The present study aimed to investigate the potential mechanism of *Dendrobium officinale (D. officinale)* on colorectal cancer and the relevant targets in the pathway using a network pharmacological approach.

**Methods:**

(1) We identified the major bioactive components of *D. officinale* by UPLC-ESI-MS/MS and established the in-house library by using the literature mining method. (2) Target prediction was performed by SwissADME and SwissTargetPrediction. (3) A protein–protein interaction (PPI) network and component–target–pathway network (C-T-P network) were constructed. (4) The GO pathways and the KEGG pathway enrichment analysis were carried out by the Metascape database. (5) Molecular docking was performed by AutoDock software. (6) A series of experimental assays including cell proliferation, cell invasion and migration, and TUNEL staining in CRC were performed in CRC cell lines (HT-29, Lovo, SW-620, and HCT-116) to confirm the inhibitory effects of *D. officinale*.

**Results:**

(1) In total, 396 candidate active components of *D. officinale* were identified by UPLC-ESI-MS/MS and selected from the database. (2) From OMIM, GeneCards, DrugBank, and TTD databases, 1,666 gene symbols related to CRC were gathered, and (3) 34 overlapping gene symbols related to CRC and drugs were obtained. (4) These results suggested that the anti-CRC components of *D. officinale* were mainly apigenin, naringenin, caffeic acid, γ-linolenic acid, α-linolenic acid, cis-10-heptadecenoic acid, etc., and the core targets of action were mainly ESR1, EGFR, PTGS2, MMP9, MMP2, PPARG, etc. (5) The proliferation of muscle cells, the regulation of inflammatory response, the response of cells to organic cyclic compounds, and the apoptotic signaling pathway might serve as principal pathways for CRC treatment. (6) The reliability of some important active components and targets was further validated by molecular docking. The molecular docking analysis suggested an important role of apigenin, naringenin, PTGS2, and MMP9 in delivering the pharmacological activity of *D. officinale* against CRC. (7) These results of the evaluation experiment *in vitro* suggested that *D. officinale* had a strong inhibitory effect on CRC cell lines, and it exerted anti-CRC activity by activating CRC cell apoptosis and inhibiting CRC cell migration and invasion.

**Conclusion:**

This study may provide valuable insights into exploring the mechanism of action of *D. officinale* against CRC.

## Introduction

Colorectal cancer (CRC) is the third most common cancer after lung and breast cancers, and it is also the second leading cause of cancer-related mortality (1). *Dendrobium officinale* Kimura & Migo (*D. officinale*), a widely known traditional food–medicine herb, has been commonly employed in China and other Asian countries for thousands of years (2). According to Shennong's *Classic of Materia Medica*, the famous classic book of Chinese materia medica, it could benefit the intestines and stomach, supplementing body fluids (3). Also, the functions of benefiting the intestines and stomach, increasing saliva, nourishing Yin, and clearing heat were recorded by Chinese pharmacopeia (2020 edition). *D. officinale* has also been widely used as a traditional Chinese medicinal herb for thousands of years to treat gastrointestinal sickness in China (4). More importantly, their significant anti-CRC benefits have also been validated (1, 5–10).

However, previous studies have shown that *D. officinale* possesses a variety of active components, including polysaccharides (1, 6, 11, 12), flavonoids (13), alkaloids (14), amino acids (15), and bibenzyl (16). The underlying mechanisms of anti-colon cancer effects of *D. officinale* remain unclear because of the complex compositions. Moreover, the action mechanism of *D. officinale* inhibits the colon cancer cell growth, and metastasis is not clear, indicating the need for an investigation.

Traditional Chinese herb medicine or formulations contain multiple active components, targets, and pathways, which are not able to be elucidated by traditional methods (17). Fortunately, the approach of network pharmacology is an effective method to analyze traditional Chinese medicine for both prescription and single herb (18, 19) and provides a new perspective for studying Chinese herbal formulas (20). As a new paradigm in drug studies, derived from systems biology and bioinformatics, network pharmacology can reveal the underlying molecular mechanisms of Chinese herb medicine, *via* the construction and visualization of “medicine–target–disease” interaction network (21, 22). Network pharmacology can reflect the “multiple components–multiple targets–multiple pathways” of anticancer effects of Chinese herb medicine (23), which is potential to solve the same problems of the *D. officinale* methanolic extract.

**Figure 1:**
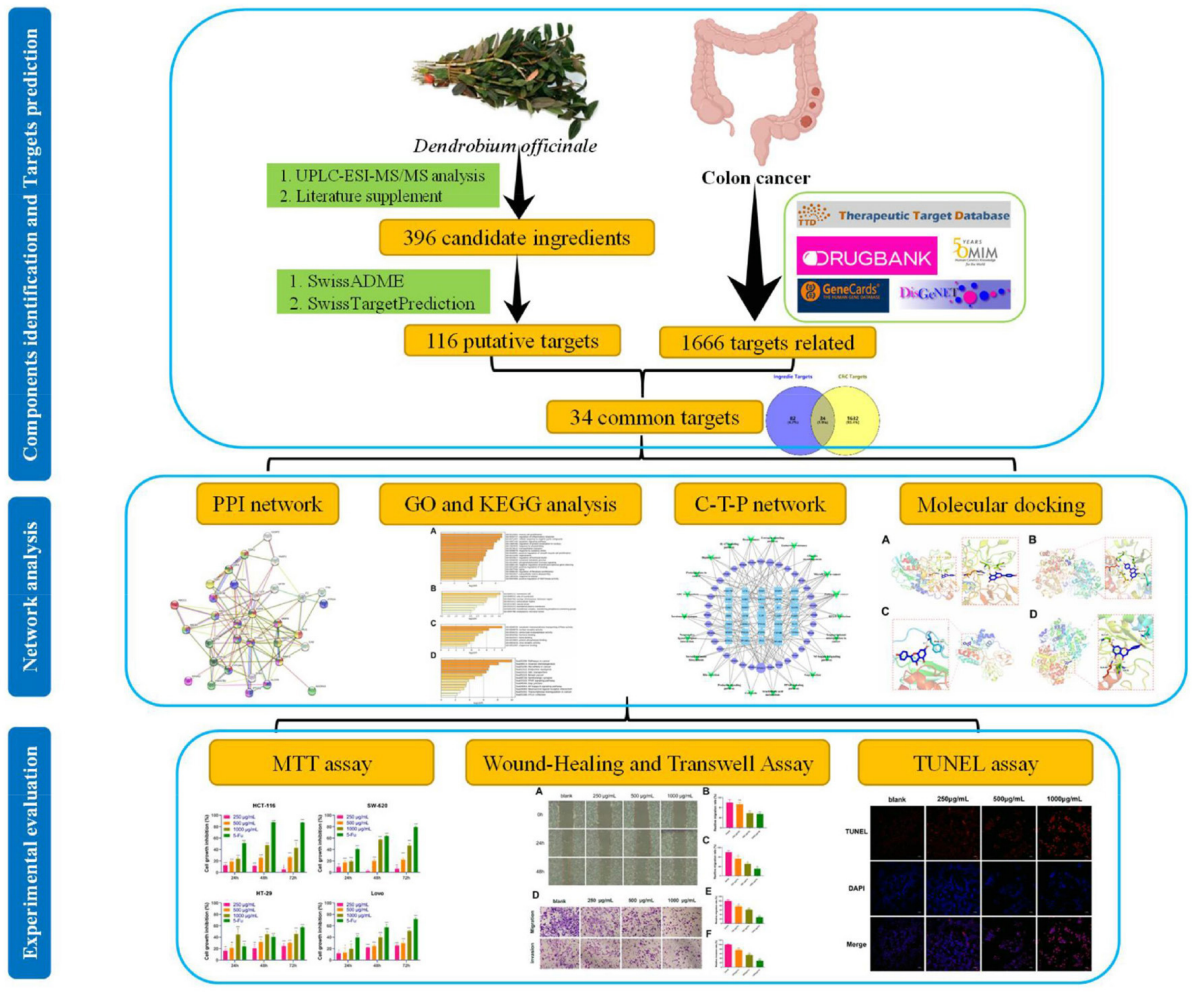
Workflow of network pharmacology analysis and validation of *D. officinale* on CRC.

This study investigated the potential mechanism of *D. officinale* on CRC and the relevant targets in the pathway. First, we identified the major bioactive components of *D. officinale* by UPLC-ESI-MS/MS and established the in-house library. Second, a series of network pharmacological analyses, including target prediction, GO and KEGG enrichment analysis, and network construction, were conducted to identify the CRC-related targets and potential mechanisms of *D. officinale*. Third, the reliability of some key active components and targets was further validated by molecular docking. Finally, a series of experimental assays including cell proliferation, cell invasion and migration, and TUNEL staining were performed in CRC cell lines to confirm the inhibitory effects of *D. officinale*. The workflow of our network pharmacological and experimental studies of *D. officinale* in CRC is shown in Figure 1. By characterizing the *D. officinale* composition and its therapeutic mechanisms on CRC, we laid the foundation for the clinical application of *D. officinale*.

## Materials and Methods

### Chemicals and Reagents

*D. officinale* was collected from Shaoguan Hejiantang Ecological Agriculture Company Ltd. (latitude 24°80' N, longitude 113°59' E, Shaoguan, Guangdong, China) in June 2020 and was identified by Professor Yuechun Huang (Laboratory of the Department of Clinical Pharmacy, College of the First Clinical Medical, Guangzhou University of Chinese Medicine). The voucher specimen number was 57826, and a specimen was deposited in the School of Pharmaceutical Sciences, Guangzhou University of Chinese Medicine (Guangzhou, China).

The human colorectal cancer (CRC) cell lines HT-29, Lovo, SW-620, and HCT-116 were obtained from the Cell Line Bank of the Chinese Academy of Sciences (Shanghai, China). Reagents 5-fluorouracil (5-Fu), dimethyl sulfoxide (DMSO), and 3-[4,5-dimethylthiazol-2-yl]-2,5-diphenyltetrazolium bromide (MTT) were purchased from Sigma Aldrich Co. (St. Louis, MO, USA). HPLC-grade acetonitrile, methanol, and acetic acid were purchased from Merck Millipore (Billerica, MA, USA). Ultrapure water was prepared using a Millipore Alpha-Q water system (Merck Millipore). All other reagents were of analytical grade; 24-well chambers with 8 μm pore size (#353097) and Matrigel matrix (#354263) were purchased from BD Biosciences (San Jose, CA, USA). Cell culture dishes and plates were purchased from Wuxi NEST biotechnology Co. (Wuxi, Jiangsu, China); 4% paraformaldehyde was purchased from Biosharp Life Sciences (Hefei, Anhui, China); 0.1% crystal violet was purchased from Beijing Leagene Biotech Co., Ltd (Beijing, China). The One Step TUNEL Apoptosis Assay kit (#C1089), Enhanced Immunostaining Permeabilization Solution (#P0097), and DAPI Staining Solution (#C1006) were purchased from Beyotime Biotechnology (Shanghai, China).

### Sample Preparation

Leaves from fresh stems of *D. officinale* were removed and cleaned with tap water. Then, the vacuum freeze-dried stems were ground to a powder (MM 400, Retsch, Haan, Germany) at 30 Hz for 1.5 min in a grinder. The powder (100 mg) was extracted for 24 h at 4°C with 0.6 ml 70% aqueous methanol and vortexed six times to increase the extraction rate. After centrifuging at 10,000 × *g* for 10 min, the supernatant was aspirated and filtered using a 0.22 μm microporous membrane for UPLC-ESI-MS/MS analysis.

### Ultra-High-Performance Liquid Chromatography and Mass Spectrometry Conditions

The data acquisition instrument system included a UPLC-ESI-MS/MS system, which mainly includes UPLC (Shim-pack UFLC SHIMADZU CBM30A, SHIMADZU, Kyoto, Japan) coupled with an ESI-triple quadrupole linear ion trap MS/MS system (Applied Biosystems 4500 Q TRAP; AB SCIEX, Foster City, CA, USA).

The filtered supernatant was separated using an Acquity UHPLC HSS T3 C18 column (2.1 mm × 100 mm, 1.8 μm; Waters, Milford, MA, USA). The mobile phase consisted of 0.04% acetic acid in deionized water (mobile phase A) and 0.04% acetic acid in acetonitrile (mobile phase B). Separation was conducted using the following gradient elution: 0–10.00 min, 5–95% B; 10.00–11.00 min, 95% B; 11.0–11.10 min, 95–5% B; and 11.10–14.00 min, 5% B for equilibration of the column. The column temperature was maintained at 40°C. The flow rate was set at 0.35 ml/min, and the injection volume was 4 μL.

The mass spectrometry conditions were listed as follows: the electrospray ionization (ESI) temperature was 550°C, the ion spray voltage (IS) was 5,500 V (positive ion mode)/−4,500 V (negative ion mode), the curtain gas (CUR) was set to 30 psi, and the collision-induced dissociation (CAD) parameter was set high. In the triple quadrupole (QQQ), declustering potential (DP) and collision energy (CE) for individual multiple reaction monitoring (MRM) transitions were done with further DP and CE optimization (24, 25). Based on the self-built MetWare database (Wuhan Metware Biotechnology Co., Ltd., Wuhan, China), the material was characterized according to the secondary spectrum information.

### Active Component Preparation From *D. officinale* and Target Prediction

The chemical composition of *D. officinale* was mainly obtained from UPLC-ESI-MS/MS analysis. Furthermore, we used the literature mining method (PubMed, CNKI.net, Cqvip.com, and Web of Science) to search for those that had been reported as the key active components of *D. officinale*, while had not been identified in UPLC-ESI-MS/MS analysis. The in-house library that covers all phytochemical constituents obtained from UPLC-ESI-MS/MS analysis and previously reported compounds from the literature is established in Table 1 and Supplementary Table 1, including the compound name, molecular weight, molecular formula, ion mode, and related references. The in-house library totally acquired 396 active components after removing duplicates.

**Table 1:** Component identification results of 70% methanol extracts from *D. officinale*.

**ID**	**Rt (min)**	**MW (g/mol)**	**Molecular Formula**	**Ionization Mode**	**Compounds**	**CAS**
**Flavonoids**						
1	3.90	624.14	C_28_H_32_O_16_	[M–H]^−^	Isorhamnetin-3-O-rutinoside	604-80-8
2	4.09	304.05	C_15_H_12_O_7_	[M–H]^−^	Taxifolin	480-18-2
3	4.28	448.08	C_21_H_20_O_11_	[M+H]^+^	Quercitrin	522-12-3
4	3.73	610.13	C_27_H_30_O_16_	[M–H]^−^	Rutin	153-18-4
5	3.65	464.08	C_21_H_20_O_12_	[M–H]^−^	Hyperin	482-36-0
6	5.81	316.05	C_16_H_12_O_7_	[M–H]^−^	Isorhamnetin	480-19-3
7	3.75	432.09	C_21_H_20_O_10_	[M+H]^+^	Apigenin 5-O-glucoside	28757-27-9
8	3.98	464.08	C_21_H_20_O_12_	[M–H]^−^	Isoquercitrin	482-35-9
9	3.75	442.07	C_22_H_18_O_10_	[M–H]^−^	(-)-Catechin gallate	130405-40-2
10	3.80	464.08	C_21_H_20_O_12_	[M–H]^−^	Spiraeoside	20229-56-5
11	5.45	272.06	C_15_H_12_O_5_	[M–H]^−^	Pinobanksin	548-82-3
12	6.19	316.05	C_16_H_12_O_7_	[M–H]^−^	Rhamnetin (7-O-Methxyl Quercetin)	90-19-7
13	5.55	302.07	C_16_H_14_O_6_	[M+H]^+^	Homoeriodictyol	446-71-9
14	6.81	286.07	C_16_H_14_O_5_	[M+H]^+^	Isosakuranetin (4'-Methylnaringenin)	480-43-3
15	4.05	580.15	C_27_H_32_O_14_	[M–H]^−^	Narirutin	14259-46-2
16	3.18	594.13	C_27_H_30_O_15_	[M+H]^+^	Apigenin 6,8-C-diglucoside	23666-13-9
17	3.52	564.12	C_26_H_28_O_14_	[M+H]^+^	Isoschaftoside	52012-29-0
18	3.46	448.08	C_21_H_20_O_11_	[M+H]^+^	Orientin	28608-75-5
19	4.00	464.08	C_21_H_20_O_12_	[M–H]^−^	Gossypitrin	491-50-9
20	3.97	450.10	C_21_H_22_O_11_	[M–H]^−^	Astilbin	29838-67-3
21	3.70	432.09	C_21_H_20_O_10_	[M–H]^−^	Isovitexin	29702-25-8
22	3.46	448.08	C_21_H_20_O_11_	[M–H]^−^	HoMoorientin	4261-42-1
23	3.34	564.12	C_26_H_28_O_14_	[M+H]^+^	Schaftoside	51938-32-0
24	4.29	303.04	C_15_H_11_O_7_	[M]^+^	Delphinidin chloride	528-53-0
25	5.97	316.05	C_16_H_12_O_7_	[M+H]^+^	Tamarixetin	603-61-2
26	3.98	301.06	C_16_H_13_O_6_	[M]^+^	Peonidin	134-01-0
27	3.38	610.13	C_27_H_30_O_16_	[M+H]^+^	Luteolin 3',7-di-O-glucoside	52187-80-1
28	5.86	330.06	C_17_H_14_O_7_	[M–H]^−^	Di-O-methylquercetin	2068/2/2
29	3.96	578.14	C_27_H_30_O_14_	[M+H]^+^	Apigenin 7-rutinoside (Isorhoifolin)	552-57-8
30	3.79	594.13	C_27_H_30_O_15_	[M–H]^−^	Kaempferol 3-O-rutinoside (Nicotiflorin)	17650-84-9
31	5.54	272.06	C_15_H_12_O_5_	[M–H]^−^	Naringenin	480-41-1
32	3.72	434.10	C_21_H_22_O_10_	[M–H]^−^	Isohemiphloin	3682-02-8
33	3.77	448.08	C_21_H_20_O_11_	[M+H]^+^	Luteolin 7-O-glucoside (Cynaroside)	5373-11-5
34	5.47	272.06	C_15_H_12_O_5_	[M+H]^+^	Naringenin chalcone	73692-50-9
35	3.81	464.08	C_21_H_20_O_12_	[M–H]^−^	Quercetin 3-O-glucoside (Isotrifoliin)	21637-25-2
36	3.49	578.14	C_27_H_30_O_14_	[M+H]^+^	Vitexin 2”-O-β-L-rhamnoside	64820-99-1
37	5.01	284.06	C_16_H_12_O_5_	[M+H]^+^	Calycosin	20575-57-9
38	5.60	272.06	C_15_H_12_O_5_	[M+H]^+^	Butin	492-14-8
39	3.56	610.13	C_27_H_30_O_16_	[M–H]^−^	Bioquercetin	52525-35-6
40	5.74	300.05	C_16_H_12_O_6_	[M+H]^+^	Hispidulin	1447-88-7
41	5.90	330.06	C_17_H_14_O_7_	[M+H]^+^	Jaceosidin	18085-97-7
42	3.86	432.09	C_21_H_20_O_10_	[M+H]^+^	Apigenin-8-C-glucoside	3681-93-4
43	3.02	610.13	C_27_H_30_O_16_	[M+H]^+^	Luteolin-6,8-di-C-glucoside	29428-58-8
44	3.73	432.09	C_21_H_20_O_10_	[M+H]^+^	Genistein 8-C-glucoside	66026-80-0
45	3.77	446.10	C_22_H_22_O_10_	[M+H]^+^	Calycosin-7-glucoside	20633-67-4
46	5.63	270.04	C_15_H_10_O_5_	[M+H]^+^	Apigenin	520-36-5
47	3.31	610.13	C_27_H_30_O_16_	[M+H]^+^	Luteolin-7,3'-Di-O-β-D-Glucoside	257-724-7
48	3.75	594.13	C_27_H_30_O_15_	[M+H]^+^	Lonicerin	20633-84-5
49	3.55	578.14	C_27_H_30_O_14_	[M+H]^+^	Violanthin	40581-17-7
**Lignans and Coumarins**						
50	5.36	358.13	C_20_H_22_O_6_	[M–H]^−^	Pinoresinol	487-36-5
51	5.74	160.05	C_10_H_8_O_2_	[M+H]^+^	6-MethylCoumarin	92-48-8
52	2.71	340.07	C_15_H_16_O_9_	[M–H]^−^	Esculin(6,7-DihydroxyCoumarin-6-glucoside)	531-75-9
53	3.01	354.08	C_16_H_18_O_9_	[M+H]^+^	Scopolin	531-44-2
54	4.00	192.04	C_10_H_8_O_4_	[M+H]^+^	Scopoletin(7-Hydroxy-5-methoxycoumarin)	92-61-5
55	4.48	162.03	C_9_H_6_O_3_	[M–H]^−^	4-Hydroxycoumarin	1076-38-6
56	3.34	682.21	C_32_H_42_O_16_	[M–H]^−^	Pinoresinol diglucoside	63902-38-5
57	4.16	550.18	C_27_H_34_O_12_	[M–H]^−^	Eucommin A	99633-12-2
58	5.21	418.14	C_22_H_26_O_8_	[M–H]^−^	(+)-Syringaresinol	21453-69-0
59	5.30	388.13	C_21_H_24_O_7_	[M–H]^−^	Medioresinol	40957-99-1
60	7.82	368.11	C_21_H_20_O_6_	[M+H]^+^	Glycycoumarin	94805-82-0
**Lipid**						
61	7.48	314.22	C_18_H_34_O_4_	[M–H]^−^	9,10-Dihydroxy-12-octadecenoic acid	263399-34-4
62	9.03	296.21	C_18_H_32_O_3_	[M–H]^−^	13-Hydroxy-9,11-octadecadienoic acid	5204-88-6
63	9.03	296.21	C_18_H_32_O_3_	[M–H]^−^	9-Hydroxy-10,12-octadecadienoic acid	15514-85-9
64	10.85	228.19	C_14_H_28_O_2_	[M–H]^−^	Myristic Acid	544-63-8
65	0.76	257.10	C_8_H_20_NO_6_P	[M+H]^+^	Choline alfoscerate	28319-77-9
66	10.02	523.33	C_26_H_54_NO_7_P	[M+H]^+^	1-Stearoyl-sn-glycero-3-phosphocholine	19420-57-6
67	8.99	479.27	C_23_H_46_NO_7_P	[M–H]^−^	LysoPE 18:1	89576-29-4
68	11.38	242.21	C_15_H_30_O_2_	[M–H]^−^	Pentadecanoic Acid	1002-84-2
69	10.85	254.21	C_16_H_30_O_2_	[M–H]^−^	Palmitoleic Acid	373-49-9
70	10.78	278.21	C_18_H_30_O_2_	[M–H]^−^	γ-Linolenic Acid	506-26-3
71	10.56	278.21	C_18_H_30_O_2_	[M–H]^−^	α-Linolenic Acid	463-40-1
72	10.96	304.22	C_20_H_32_O_2_	[M–H]^−^	Arachidonic Acid	506-32-1
73	10.81	328.22	C_22_H_32_O_2_	[M–H]^−^	Cis-4,7,10,13,16,19-Docosahexaenoic Acid(C22:6n3)	6217-54-5
74	11.47	268.22	C_17_H_32_O_2_	[M–H]^−^	Cis-10-Heptadecenoic Acid	29743-97-3
75	12.11	282.23	C_18_H_34_O_2_	[M–H]^−^	Elaidic Acid	112-79-8
76	9.14	186.15	C_11_H_22_O_2_	[M–H]^−^	Undecylic Acid	112-37-8
77	7.19	272.21	C_16_H_32_O_3_	[M–H]^−^	16-Hydroxy hexadecanoic acid	506-13-8
78	11.05	284.25	C_18_H_36_O_2_	[M–H]^−^	Stearic Acid	57-11-4
79	11.90	282.23	C_18_H_34_O_2_	[M–H]^−^	11-Octadecanoic acid(Vaccenic acid)	506-17-2
80	6.53	216.16	C_12_H_24_O_3_	[M–H]^−^	12-Hydroxydodecanoic acid	505-95-3
81	8.74	495.30	C_24_H_50_NO_7_P	[M+H]^+^	LysoPC 16:0	17364-16-8
82	8.68	453.25	C_21_H_44_NO_7_P	[M+H]^+^	LysoPE 16:0	53862-35-4
83	8.91	278.21	C_18_H_30_O_2_	[M+H]^+^	Punicic acid	544-72-9
84	9.62	200.16	C_12_H_24_O_2_	[M–H]^−^	Lauric acid	143-07-7
85	8.92	296.21	C_18_H_32_O_3_	[M–H]^−^	9,10-EODE	65167-83-1
86	8.37	294.20	C_18_H_30_O_3_	[M–H]^−^	9-HOTrE	89886-42-0
87	9.12	294.20	C_18_H_30_O_3_	[M–H]^−^	9-KODE	54232-59-6
88	7.77	294.20	C_18_H_30_O_3_	[M–H]^−^	13-HOTrE(r)	74784-20-6
89	9.70	296.21	C_18_H_32_O_3_	[M–H]^−^	12,13-EODE	6799-85-5
90	11.01	310.26	C_20_H_38_O_2_	[M–H]^−^	Eicosenoic acid	26764-41-0
91	12.09	308.25	C_20_H_36_O_2_	[M–H]^−^	Eicosadienoic acid	5598-38-9
92	6.14	288.21	C_16_H_32_O_4_	[M–H]^−^	10,16-Dihydroxy-palmitic acid	3233-90-7
93	9.23	354.25	C_21_H_38_O_4_	[M+H]^+^	Glyceryl linoleate	26545-74-4
94	9.68	330.25	C_19_H_38_O_4_	[M+H]^+^	Glycerin Monopalmitate	542-44-9
95	7.30	301.27	C_18_H_39_NO_2_	[M+H]^+^	D-erythro-Dihydrosphingosine	764-22-7
**Amino acids and their derivatives**						
96	0.89	144.09	C_7_H_14_NO${}_{2}^{+}$	[M]^+^	Proline βine (ProBet)	1195-94-4
97	0.91	129.07	C_6_H_11_NO_2_	[M+H]^+^	Pipecolic acid	4043-87-2
98	0.89	143.09	C_7_H_13_NO_2_	[M+H]^+^	1,2-N-Methylpipecolic acid	7730-87-2
99	0.74	132.05	C_4_H_8_N_2_O_3_	[M+H]^+^	L-Asparagine Anhydrous	70-47-3
100	0.76	131.05	C_5_H_9_NO_3_	[M+H]^+^	Trans-4-Hydroxy-L-proline	51-35-4
101	0.71	133.03	C_4_H_7_NO_4_	[M–H]^−^	L-Aspartic Acid	56-84-8
102	0.77	240.01	C_6_H_12_N_2_O_4_S_2_	[M+H]^+^	L-(-)-Cystine	56-89-3
103	1.20	131.09	C_6_H_13_NO_2_	[M+H]^+^	L-Leucine	61-90-5
104	1.13	181.07	C_9_H_11_NO_3_	[M+H]^+^	L-(-)-Tyrosine	60-18-4
105	0.68	155.06	C_6_H_9_N_3_O_2_	[M+H]^+^	L-Histidine	71-00-1
106	0.84	117.07	C_5_H_11_NO_2_	[M+H]^+^	L-Valine	72-18-4
107	1.23	131.09	C_6_H_13_NO_2_	[M+H]^+^	L-Isoleucine	73-32-5
108	0.73	174.10	C_6_H_14_N_4_O_2_	[M+H]^+^	L-(+)-Arginine	74-79-3
109	0.67	169.08	C_7_H_11_N_3_O_2_	[M+H]^+^	3-N-Methyl-L-histidine	368-16-1
110	2.47	204.08	C_11_H_12_N_2_O_2_	[M–H]^−^	L-Tryptophan	73-22-3
111	2.49	280.09	C_13_H_16_N_2_O_5_	[M+H]^+^	Asp-phe	13433-09-5
112	3.52	312.13	C_18_H_20_N_2_O_3_	[M+H]^+^	Phe-Phe	2577-40-4
113	1.92	188.10	C_8_H_16_N_2_O_3_	[M+H]^+^	N-Glycyl-L-leucine	869-19-2
114	1.24	129.04	C_5_H_7_NO_3_	[M–H]^−^	5-Oxoproline	149-87-1
115	0.76	105.04	C_3_H_7_NO_3_	[M–H]^−^	D-Serine	312-84-5
116	0.75	303.13	C_12_H_21_N_3_O_6_	[M+H]^+^	Nicotianamine	34441-14-0
117	0.79	131.05	C_5_H_9_NO_3_	[M+H]^+^	Cis-4-Hydroxy-D-proline	2584-71-6
118	1.35	131.09	C_6_H_13_NO_2_	[M+H]^+^	α-Aminocaproic acid	327-57-1
119	1.25	612.12	C_20_H_32_N_6_O_12_S_2_	[M–H]^−^	Oxidized Glutathione	121-24-4
120	2.29	236.10	C_12_H_16_N_2_O_3_	[M+H]^+^	DL-Alanyl-DL-phenylalanine	1999-45-7
121	3.54	278.15	C_15_H_22_N_2_O_3_	[M+H]^+^	Leucylphenylalanine	56217-82-4
122	1.86	188.10	C_8_H_16_N_2_O_3_	[M+H]^+^	Glycylisoleucine	19461-38-2
123	2.26	222.09	C_11_H_14_N_2_O_3_	[M+H]^+^	Glycylphenylalanine	721-66-4
124	3.83	246.09	C_13_H_14_N_2_O_3_	[M–H]^−^	Acetyltryptophan	2280-01-5
125	0.79	115.06	C_5_H_9_NO_2_	[M+H]^+^	L-Proline	147-85-3
126	0.76	175.08	C_6_H_13_N_3_O_3_	[M+H]^+^	L-Citrulline	372-75-8
127	0.83	147.05	C_5_H_9_NO_4_	[M+H]^+^	L-Glutamic acid	56-86-0
128	0.68	146.10	C_6_H_14_N_2_O_2_	[M+H]^+^	L-(+)-Lysine	56-87-1
129	1.22	211.07	C_10_H_13_NO_4_	[M+H]^+^	(-)-3-(3,4-Dihydroxyphenyl)-2-methylalanine	555-30-6
130	1.02	188.10	C_8_H_16_N_2_O_3_	[M+H]^+^	N6-Acetyl-L-lysine	692-04-6
131	0.75	160.08	C_6_H_12_N_2_O_3_	[M–H]^−^	D-Alanyl-D-Alanine	923-16-0
132	1.17	188.07	C_7_H_12_N_2_O_4_	[M–H]^−^	N-α-Acetyl-L-glutamine	2490-97-3
133	1.12	216.11	C_8_H_16_N_4_O_3_	[M+H]^+^	N-α-Acetyl-L-arginine	155-84-0
134	0.72	169.08	C_7_H_11_N_3_O_2_	[M+H]^+^	1-Methylhistidine	332-80-9
135	0.68	146.06	C_5_H_10_N_2_O_3_	[M+H]^+^	L-Glutamine	56-85-9
136	0.73	190.08	C_7_H_14_N_2_O_4_	[M+H]^+^	2,6-Diaminooimelic acid	583-93-7
137	1.20	137.08	C_8_H_11_NO	[M+H]^+^	L-Tyramine	51-67-2
138	1.14	307.07	C_10_H_17_N_3_O_6_S	[M–H]^−^	Glutathione reduced form	70-18-8
139	1.18	149.04	C_5_H_11_NO_2_S	[M+H]^+^	L-Methionine	63-68-3
140	1.58	220.08	C_11_H_12_N_2_O_3_	[M+H]^+^	5-Hydroxy-L-tryptophan	56-69-9
141	0.84	117.07	C_5_H_11_NO_2_	[M+H]^+^	DL-Norvaline	760-78-1
142	1.40	162.04	C_6_H_10_O_5_	[M–H]^−^	3-Hydroxy-3-methylpentane-1,5-dioic acid	503-49-1
143	0.74	103.06	C_4_H_9_NO_2_	[M+H]^+^	2-Aminoisobutyric acid	62-57-7
144	0.75	103.06	C_4_H_9_NO_2_	[M+H]^+^	N,N-Dimethylglycine	1118-68-9
145	0.68	188.11	C_7_H_16_N_4_O_2_	[M+H]^+^	H-HomoArg-OH	156-86-5
146	1.15	197.06	C_9_H_11_NO_4_	[M+H]^+^	3,4-Dihydroxy-DL-phenylalanine	63-84-3
147	3.90	290.11	C_15_H_18_N_2_O_4_	[M–H]^−^	N-Acetyl-DL-tryptophan	87-32-1
148	1.91	165.07	C_9_H_11_NO_2_	[M+H]^+^	Phenylalanine	63-91-2
**Alkaloids**						
149	0.60	202.20	C_10_H_26_N_4_	[M+H]^+^	Spermine	71-44-3
150	0.78	117.07	C_5_H_11_NO_2_	[M+H]^+^	Betaine	107-43-7
151	3.79	550.12	C_24_H_26_N_2_O_13_	[M+H]^+^	Batanin	7659-95-2
152	0.80	161.06	C_6_H_11_NO_4_	[M+H]^+^	DL-2-Aminoadipic acid	542-32-5
153	0.84	153.07	C_8_H_11_NO_2_	[M+H]^+^	Dopamine hydrochloride	62-31-7
154	5.77	270.08	C_16_H_14_O_4_	[M+H]^+^	Isomethacin	74560-05-7
155	1.89	135.05	C_5_H_5_N_5_	[M+H]^+^	Aminopurine	452-06-2
156	0.74	103.09	C_5_H_13_NO	[M+H]^+^	Choline	62-49-7
157	2.27	234.12	C_13_H_18_N_2_O_2_	[M+H]^+^	N-p-Coumaroyl putrescine	34136-53-3
158	0.64	130.11	C_5_H_14_N_4_	[M+H]^+^	Agmatine	306-60-5
159	2.47	117.05	C_8_H_7_N	[M+H]^+^	Indole	120-72-9
160	2.42	473.14	C_20_H_23_N_7_O_7_	[M+H]^+^	10-Formyl-THF	2800-34-2
161	6.53	342.26	C_19_H_38_N_2_O_3_	[M+H]^+^	Cocamidopropyl βine	86438-79-1
162	0.79	137.04	C_7_H_7_NO_2_	[M+H]^+^	Trigonelline	535-83-1
163	0.72	130.10	C_6_H_14_N_2_O	[M+H]^+^	N-Acetylputrescine	18233-70-0
164	6.09	350.02	C_16_H_11_N_2_NaO_4_S	[M–Na]^−^	Shikonin	523-44-4
165	9.01	516.31	C_33_H_44_N_2_O_3_	[M+CH_3_COOH-H]^−^	Dendrocrepine	51020-39-4
166	9.19	516.31	C_33_H_44_N_2_O_3_	[M+CH_3_COOH-H]^−^	Isodendrocrepine	50906-94-0
167	2.11	264.17	C_15_H_24_N_2_O_2_	[M+H]^+^	Sophoranol	3411-37-8
168	1.20	131.09	C_6_H_13_NO_2_	[M+H]^+^	6-Deoxyfagomine	197449-09-5
169	2.94	310.15	C_16_H_24_NO${}_{5}^{+}$	[M]^+^	Sinapine	18696-26-9
170	2.88	280.14	C_15_H_22_NO${}_{4}^{+}$	[M]^+^	Feruloylcholine	85927-25-9
171	4.79	283.11	C_17_H_17_NO_3_	[M+H]^+^	N-p-Coumaroyltyramine	36417-86-4
172	6.03	624.22	C_36_H_36_N_2_O_8_	[M+H]^+^	Cannabisin F	163136-19-4
173	4.76	313.13	C_18_H_19_NO_4_	[M+H]^+^	N-Trans-feruloyltyramine	66648-43-9
174	4.91	313.13	C_18_H_19_NO_4_	[M+H]^+^	N-Cis-feruloyltyramine	80510-09-4
**Phenolic acids**						
175	4.03	194.05	C_10_H_10_O_4_	[M–H]^−^	Ferulic acid	1135-24-6
176	3.38	198.04	C_9_H_10_O_5_	[M–H]^−^	Syringic acid	530-57-4
177	3.29	168.04	C_8_H_8_O_4_	[M–H]^−^	Vanillic acid	121-34-6
178	3.86	180.07	C_10_H_12_O_3_	[M–H]^−^	Coniferyl alcohol	458-35-5
179	4.09	152.04	C_8_H_8_O_3_	[M–H]^−^	2-Methoxybenzoic acid	529-75-9
180	2.74	354.08	C_16_H_18_O_9_	[M–H]^−^	Chlorogenic acid	327-97-9
181	3.97	152.04	C_8_H_8_O_3_	[M–H]^−^	Vanillin	121-33-5
182	3.69	122.03	C_7_H_6_O_2_	[M–H]^−^	4-Hydroxybenzaldehyde	123-08-0
183	3.05	138.03	C_7_H_6_O_3_	[M–H]^−^	4-Hydroxybenzoic acid	99-96-7
184	3.83	210.08	C_11_H_14_O_4_	[M–H]^−^	Sinapyl alcohol	537-33-7
185	2.85	154.02	C_7_H_6_O_4_	[M–H]^−^	2,4-Dihydroxy benzoic acid	89-86-1
186	2.72	342.11	C_16_H_22_O_8_	[M–H]^−^	Coniferin	531-29-3
187	3.31	786.22	C_35_H_46_O_20_	[M–H]^−^	Echinacoside	82854-37-3
188	4.28	666.18	C_31_H_38_O_16_	[M–H]^−^	2'-Acetylacteoside	94492-24-7
189	5.53	178.06	C_10_H_10_O_3_	[M–H]^−^	Trans-4-Hydroxycinnamic Acid Methyl Ester	19367-38-5
190	4.10	182.05	C_9_H_10_O_4_	[M–H]^−^	Syringic Aldehyde	134-96-3
191	4.09	194.05	C_10_H_10_O_4_	[M–H]^−^	Trans-ferulic acid	537-98-4
192	3.14	180.04	C_9_H_8_O_4_	[M–H]^−^	Caffeic acid	331-39-5
193	5.22	148.05	C_9_H_8_O_2_	[M–H]^−^	Cinnamic acid	140-10-3
194	3.05	138.06	C_8_H_10_O_2_	[M–H]^−^	Tyrosol	501-94-0
195	4.01	224.06	C_11_H_12_O_5_	[M–H]^−^	Sinapic acid	530-59-6
196	3.15	386.10	C_17_H_22_O_10_	[M–H]^−^	1-O-β-D-Glucopyranosyl sinapate	78185-48-5
197	3.79	164.04	C_9_H_8_O_3_	[M+H]^+^	p-Coumaric acid	501-98-4
198	4.43	208.06	C_11_H_12_O_4_	[M–H]^−^	Sinapinaldehyde	4206-58-0
199	2.30	316.10	C_14_H_20_O_8_	[M–H]^−^	Cimidahurinine	142542-89-0
200	2.45	154.02	C7H_6_O_4_	[M–H]^−^	Gentisic acid	490-79-9
201	11.72	256.22	C_16_H_32_O_2_	[M–H]^−^	Hexadecanoic acid	1957/10/3
202	2.38	296.04	C_13_H_12_O_8_	[M–H]^−^	Cis-Coutaric acid	27174-07-8
203	2.87	354.08	C_16_H_18_O_9_	[M+H]^+^	4-Caffeoylquinic acid	905-99-7
204	9.71	148.01	C_8_H_4_O_3_	[M+H]^+^	Phthalic anhydride	85-44-9
205	1.75	170.02	C_7_H_6_O_5_	[M–H]^−^	Gallic acid	149-91-7
206	2.55	154.02	C_7_H_6_O_4_	[M–H]^−^	Protocatechuic acid	99-50-3
**Nucleotides and their derivatives**						
207	1.19	244.06	C_9_H_12_N_2_O_6_	[M–H]^−^	Uridine	58-96-8
208	0.75	111.04	C_4_H_5_N_3_O	[M+H]^+^	Cytosine	71-30-7
209	1.13	125.05	C_5_H_7_N_3_O	[M+H]^+^	5-Methylcytosine	554-01-8
210	1.37	345.03	C_10_H_12_N_5_O_7_P	[M–H]^−^	Guanosine 3',5'-cyclic monophosphate	7665-99-8
211	1.77	284.06	C_10_H_12_N_4_O_6_	[M–H]^−^	Xanthosine	146-80-5
212	0.79	334.04	C_11_H_15_N_2_O_8_P	[M+H]^+^	β-Nicotinamide mononucleotide	1094-61-7
213	1.84	329.04	C_10_H_12_N_5_O_6_P	[M–H]^−^	Cyclic AMP	60-92-4
214	1.43	268.07	C_10_H_12_N_4_O_5_	[M–H]^−^	9-(β-D-Arabinofuranosyl) hypoxanthine	7013-16-3
215	1.14	663.08	C_21_H_27_N_7_O_14_P_2_	[M+H]^+^	Nicotinic acid adenine dinucleotide	53-84-9
216	1.14	347.05	C_10_H_14_N_5_O_7_P	[M+H]^+^	Adenosine 5'-monophosphate	61-19-8
217	1.12	566.03	C_15_H_24_N_2_O_17_P_2_	[M–H]^−^	Uridine 5'-diphospho-D-glucose	133-89-1
218	1.13	136.03	C_5_H_4_N_4_O	[M+H]^+^	Hypoxanthine	68-94-0
219	1.12	135.05	C_5_H_5_N_5_	[M+H]^+^	Adenine	73-24-5
220	0.85	151.04	C_5_H_5_N_5_O	[M+H]^+^	2-Hydroxy-6-aminopurine	3373-53-3
221	1.88	267.08	C_10_H_13_N_5_O_4_	[M+H]^+^	Adenosine	58-61-7
222	2.08	242.08	C_10_H_14_N_2_O_5_	[M+H]^+^	Thymidine	50-89-5
223	1.15	151.04	C_5_H_5_N_5_O	[M+H]^+^	Guanine	73-40-5
224	1.37	136.03	C_5_H_4_N_4_O	[M+H]^+^	Allopurinol	315-30-0
225	1.46	283.08	C_10_H_13_N_5_O_5_	[M+H]^+^	Guanosine	118-00-3
226	1.68	267.08	C_10_H_13_N_5_O_4_	[M+H]^+^	Deoxyguanosine	961-07-9
227	1.13	227.08	C_9_H_13_N_3_O_4_	[M+H]^+^	Deoxycytidine	951-77-9
228	2.85	297.08	C_11_H_15_N_5_O_3_S	[M+H]^+^	5'-Deoxy-5'-(methylthio)adenosine	2457-80-9
229	0.68	403.99	C_9_H_14_N_2_O_12_P_2_	[M–H]^−^	Uridine 5'-diphosphate	27821-45-0
230	1.14	331.05	C_10_H_14_N_5_O_6_P	[M+H]^+^	2'-Deoxyadenosine-5'-monophosphate	653-63-4
231	1.23	324.02	C_9_H_13_N_2_O_9_P	[M–H]^−^	Uridine 5'-monophosphate	58-97-9
232	2.17	383.09	C_14_H_17_N_5_O_8_	[M+H]^+^	N6-Succinyl Adenosine	4542-23-8
233	0.82	243.07	C_9_H_13_N_3_O_5_	[M+H]^+^	Cytidine	65-46-3
234	1.96	251.09	C_10_H_13_N_5_O_3_	[M+H]^+^	Deoxyadenosine	958-09-8
235	2.23	311.11	C_12_H_17_N_5_O_5_	[M+H]^+^	2-(Dimethylamino)guanosine	2140-67-2
**Organic acid**						
236	2.09	118.06	C_5_H_10_O_3_	[M–H]^−^	3-Hydroxy-3-methyl butyric acid	625-08-1
237	0.94	174.04	C_7_H_10_O_5_	[M–H]^−^	Shikimic acid	138-59-0
238	1.31	118.02	C_4_H_6_O_4_	[M–H]^−^	Succinic acid	110-15-6
239	4.43	188.09	C_9_H_16_O_4_	[M–H]^−^	Anchoic Acid	123-99-9
240	0.97	134.02	C_4_H_6_O_5_	[M–H]^−^	L-(-)-Malic acid	636-61-3
241	1.13	192.05	C_7_H_12_O_6_	[M–H]^−^	Kinic acid	77-95-2
242	0.84	192.02	C_6_H_8_O_7_	[M–H]^−^	Citric Acid	77-92-9
243	0.86	138.04	C_6_H_6_N_2_O_2_	[M–H]^−^	UROCANIC ACID(RG)	104-98-3
244	0.95	166.04	C_5_H_10_O_6_	[M–H]^−^	D-Xylonic acid	526-91-0
245	1.11	116.01	C_4_H_4_O_4_	[M–H]^−^	Fumaric acid	110-17-8
246	1.32	118.02	C_4_H_6_O_4_	[M–H]^−^	Methylmalonic acid	516-05-2
247	2.08	132.04	C_5_H_8_O_4_	[M–H]^−^	2-Methylsuccinic acid	498-21-5
248	6.12	230.14	C_12_H_22_O_4_	[M–H]^−^	Dodecanedioic aicd	693-23-2
249	0.77	145.08	C_5_H_11_N_3_O_2_	[M+H]^+^	4-Guanidinobutyric acid	463-00-3
250	2.64	154.02	C_7_H_6_O_4_	[M–H]^−^	2,3-Dihydroxybenzoic Acid	303-38-8
251	2.63	132.07	C_6_H_12_O_3_	[M–H]^−^	5-Hydroxyhexanoic acid	185956-02-9
252	5.02	202.11	C_10_H_18_O_4_	[M–H]^−^	Sebacate	111-20-6
253	1.00	131.09	C_6_H_13_NO_2_	[M+H]^+^	6-Aminocaproic acid	60-32-2
254	0.59	174.01	C_6_H_6_O_6_	[M+H]^+^	Trans-Citridic acid	4023-65-8
255	0.77	103.06	C_4_H_9_NO_2_	[M+H]^+^	γ-Aminobutyric acid	56-12-2
256	0.69	142.02	C_6_H_6_O_4_	[M–H]^−^	Trans,trans-Muconic acid	3588-17-8
**Terpenes**						
257	8.42	236.16	C_15_H_24_O_2_	[M+H]^+^	Prehelminthosporol	1619-13-2
258	11.20	286.21	C_20_H_30_O	[M–H]^−^	Ferruginol	514-62-5
259	8.02	488.32	C_30_H_48_O_5_	[M–H]^−^	Rutundic acid	20137-37-5
260	2.42	376.12	C_16_H_24_O_10_	[M+NH_4_]^+^	Adoxosidic acid	84375-46-2
**Others**						
261	1.49	122.04	C_6_H_6_N_2_O	[M+H]^+^	Nicotinamide	98-92-0
262	0.76	180.05	C_6_H_12_O_6_	[M–H]^−^	D-(+)-Glucose	50-99-7
263	0.77	152.06	C_5_H_12_O_5_	[M–H]^−^	Ribitol	488-81-3
264	0.74	182.07	C_6_H_14_O_6_	[M–H]^−^	D-Sorbitol	50-70-4
265	3.21	376.12	C_17_H_20_N_4_O_6_	[M+H]^+^	Riboflavin	83-88-5
266	0.72	342.10	C_12_H_22_O_11_	[M–H]^−^	D-(+)-TrehaloseAnhydrous	99-20-7
267	0.73	152.06	C_5_H_12_O_5_	[M–H]^−^	D-Arabitol	488-82-4
268	0.76	152.06	C_5_H_12_O_5_	[M–H]^−^	L-Arabitol	7643-75-6
269	2.39	121.08	C_8_H_11_N	[M+H]^+^	Phenethylamine	64-04-0
270	0.71	141.01	C_2_H_8_NO_4_P	[M–H]^−^	O-Phosphorylethanolamine	1071-23-4
271	0.69	260.02	C_6_H_13_O_9_P	[M–H]^−^	D-Glucose 6-phosphate	56-73-5
272	10.80	325.27	C_20_H_39_NO_2_	[M+H]^+^	N-Oleoylethanolamine	111-58-0
273	2.75	278.11	C_11_H_22_N_2_O_4_S	[M–H]^−^	(R)-Pantetheine	496-65-1
274	0.75	342.10	C_12_H_22_O_11_	[M–H]^−^	Galactinol	3687-64-7
275	0.78	260.02	C_6_H_13_O_9_P	[M–H]^−^	Glucose-1-phosphate	59-56-3
276	0.78	182.07	C_6_H_14_O_6_	[M–H]^−^	Mannitol	87-78-5
277	0.73	342.10	C_12_H_22_O_11_	[M–H]^−^	Melibiose	585-99-9
278	2.28	219.10	C_9_H_17_NO_5_	[M+H]^+^	D-Pantothenic Acid	79-83-4
279	6.34	214.09	C_14_H_14_O_2_	[M–H]^−^	Dihydropinosylvin	14531-52-3
280	0.80	504.14	C_18_H_32_O_16_	[M–H]^−^	Panose	33401-87-5
281	0.69	422.06	C_12_H_23_O_14_P	[M–H]^−^	Trehalose 6-phosphate	4484-88-2
282	0.82	221.08	C_8_H_15_NO_6_	[M+H]^+^	N-Acetyl-D-galactosamine	1811-31-0
283	0.89	176.02	C_6_H_8_O_6_	[M–H]^−^	D-Glucurono-6,3-lactone	32449-92-6
284	0.82	342.10	C_12_H_22_O_11_	[M–H]^−^	Isomaltulose	13718-94-0
285	0.86	364.08	C_12_H_21_O_11_Na	[M+H]^+^	Turanose	547-25-1
286	1.11	123.03	C_6_H_5_NO_2_	[M+H]^+^	Nicotinic acid	59-67-6
287	0.77	152.06	C_5_H_12_O_5_	[M–H]^−^	Xylitol	87-99-0
288	0.74	180.05	C_6_H_12_O_6_	[M–H]^−^	Inositol	87-89-8
289	0.78	342.10	C_12_H_22_O_11_	[M–H]^−^	D-(+)-Sucrose	57-50-1
290	0.70	196.05	C_6_H_12_O_7_	[M–H]^−^	Gluconic acid	526-95-4
291	1.31	120.06	C_7_H_8_N_2_	[M+H]^+^	Benzamidine	618-39-3
292	2.29	205.12	C_9_H_19_NO_4_	[M+H]^+^	Pantothenol	16485-10-2
293	1.11	169.07	C_8_H_11_NO_3_	[M+H]^+^	Pyridoxine	65-23-6
294	0.75	182.07	C_6_H_14_O_6_	[M–H]^−^	Dulcitol	608-66-2
295	0.81	178.04	C_6_H_10_O_6_	[M–H]^−^	L-Gulonic-γ-lactone	1128-23-0
296	1.67	183.05	C_8_H_9_NO_4_	[M+H]^+^	4-Pyridoxic acid	82-82-6
297	4.53	188.09	C_9_H_16_O_4_	[M–H]^−^	Eucommiol	55930-44-4
298	4.68	228.07	C_14_H_12_O_3_	[M–H]^−^	9,10-Dihydrophenanthrene	776-35-2
299	3.55	410.17	C_21_H_30_O_8_	[M+H]^+^	Onitin-2'-O-D-glucoside	76947-60-9
300	6.10	242.08	C_15_H_14_O_3_	[M+H]^+^	Hircinol	41060-05-3
301	0.83	150.04	C_5_H_10_O_5_	[M–H]^−^	D-(-)-Arabinose	10323-20-3
302	6.76	270.04	C_15_H_10_O_5_	[M+H]^+^	Phosphoenolpyruvate	481-72-1
303	3.37	578.12	C_30_H_26_O_12_	[M+H]^+^	Procyanidin B3	23567-23-9
304	3.93	496.27	C_27_H_44_O_8_	[M+H]^+^	Podecdysone C	19458-46-9

The molecular structures of the compounds were downloaded from the PubChem database (https://pubchem.ncbi.nlm.nih.gov/) or mapped with ChemDraw 18.0 software and were saved in the MDL sdf. format. All chemical structures were converted to the Mol2 format using ChemBio3D Ultra software and then were prepared and converted into SMILES files using Open Babel GUI software for subsequent analyses.

The SwissADME tool (http://www.swissadme.ch/) was used for analyzing active components with absorption, distribution, metabolism, and excretion (ADME) properties and druglikeness evaluation to screen for active components with potential therapeutic effects. The screening criterion we applied in this research were (1) pharmacokinetics “high” and (2) druglikeness (DL) with more than two “yes.”

To obtain the targets of active components in *D. officinale*, SwissTargetPrediction (http://www.swisstargetprediction.ch/) (26) was employed to identify the targets. Finally, we totally acquired 116 genes after removing duplicates.

The target prediction results were sorted from high to low according to “probability,” and a threshold (probability >0.5) was set to obtain more credible targets. The official names of drug targets were retrieved through the UniProtKB search function in the UniProt database (http://www.uniprot.org/).

### Collection of Predicted Targets Related to Colon Cancer

“Colon cancer” was used as the keyword to obtain colon cancer-related targets. The known targets associated with colon cancer were derived from five databases, DisGeNet (https://www.disgenet.org/), Online Mendelian Inheritance in Man (OMIM, http://www.omim.org/), Therapeutic Target Database (TTD, http://database.idrb.cqu.edu.cn/TTD/), GeneCards (https://www.genecards.Org/), and DrugBank (http://www.drugbank.ca/). We searched these databases and totally acquired 1,666 genes after removing duplicates.

### Protein–Protein Interaction (PPI) Network Construction and Analysis

To obtain overlapping targets, VENNY 2.1 (http://bioinfogp.cnb.csic.es/tools/venny/index.html) software was used to cross *D. officinale* active component targets with CRC-related targets. Then, network protein–protein interaction network was constructed by the Search Tool for the Retrieval of Interacting Genes/Proteins (STRING) online database (https://string-db.org/, version. 11.0), using the overlap targets between *D. officinale* active components targets and CRC-related targets (27), where the species was limited to “*Homo sapiens*,” medium confidence of protein interaction data with a score > 0.400, and other basic settings were the default value.

To clarify the signaling pathways and function enrichment of potential target genes, Gene Ontology (GO) biological process enrichment analysis and Kyoto Encyclopedia of Genes and Genomes (KEGG) pathway enrichments analysis were conducted based on the Metascape database (http://metascape.org/gp/index.html) (28), using *P* < 0.01, min overlap genes = 3, and min enrichment factor > 1.5 as the cutoff criterion.

The PPI network was visualized utilizing Cytoscape v3.7.2 (www.cytoscape.org/) for further analyses (29, 30). The parameters “betweenness centrality (BC),” “closeness centrality (CC),” and “degree” were calculated using Network Analyzer to assess the topological importance of the nodes in the PPI network (31) and visualized the “component–target–pathway” network with the Merge feature of Cytoscape v3.7.2.

### Molecular Docking

To evaluate the predicted targets, AutoDock software (4.2 version) was used to dock the structures of six active components, including apigenin, naringenin, caffeic acid, γ-linolenic acid, α-linolenic acid, and cis-10-heptadecenoic acid. These active components were docked with six candidate proteins, including ESR1 (PDB ID: 1hcq), EGFR (PDB ID: 5wb7), PTGS2 (PDB ID: 5f19), MMP9 (PDB ID: 1l6j), MMP2 (PDB ID: 1eak), and PPARG (PDB ID: 3e00), respectively. The 3D structures of ligands were obtained from the ZINC database (http://zinc.docking.org/) and saved as a mol2 file. The 3D crystal structures of the candidate protein were downloaded from the RCSB Protein Data Bank (PDB) database (https://www.rcsb.org/) and saved in the pdb format.

PyMOL software separated the original ligand from the target protein and removed the water molecule, phosphate, and other inactive ligands of the target protein (32). The protein and active components were saved in the pdb format. The AutoDockTools 1.5.6 was used to convert the pdb format of the active components and proteins to the pdbqt format. The active pocket parameters were set, and AutoDock was administered for docking. When the binding energy was ≤ – 5.0 kJ/mol, the active component was considered to have good target binding activity (33).

### Experimental Evaluation

#### Preparation of *D. officinale* Methanol Aqueous Extract

To prepare the DOME, the powdered samples (100 g) of dried stems of *D. officinale* were extracted with 2 L 70% methanol three times, each time for 2 h. The methanol extract was concentrated using a rotary evaporator, and the concentrate was freeze-dried under vacuum at −20°C by using a vacuum freeze dryer (SCIENTZ-10Z, Ningbo Scientz Biotechnology Co., Ltd., Ningbo, Zhejiang, China). Finally, the DOME was dissolved in DMSO to adjust the stock concentration (20%, w/v) and stored at −20°C for future study.


$$\begin{array}{l} \text{Relative migration rate of }24\text{h }\left(\text{\%}\right)=\frac{1-\text{treated group width at }24\text{ h}/\text{treated group width at }0\text{ h}}{1-\text{ blank group width at }24\text{ h }/\text{blank group width at }0\text{ h}}\times 100\text{\%}\\ \text{Relative migration rate of }48\text{h }\left(\text{\%}\right)=\frac{1-\text{treated group width at }48\text{ h}/\text{treated group width at }0\text{ h}}{1-\text{ blank group width at }48\text{ h }/\text{blank group width at }0\text{ h}}\times 100\text{\%} \end{array}$$

#### Cell Viability

The CRC cell lines (HT-29, Lovo, SW-620, and HCT-116) were cultured in McCoy's 5A medium supplemented with 10% FBS, 100 U/ml penicillin, and 100 μg/ml streptomycin. All cultures were incubated at 37°C in an incubator with 5% CO_2_.

The effect of the DOME on HT-29, Lovo, SW-620, and HCT-116 cell growth was assessed using an MTT assay. In brief, the cells in the logarithmic phase were seeded in 96-well plates at 1 × 10^4^ cells/well. After incubation for 24 h, they were exposed to the DOME (0, 250, 500, and 1,000 μg/ml) for another 24, 48, and 72 h in triplicate. Then 5-Fu (50 μg/mL) was used as the positive control, 20 μL MTT solution (5 mg/ml) was added to each well, and the cells were incubated for 4 h at 37°C in an incubator with 5% CO_2_. After discarding the supernatants, 100 μL DMSO was added, and the absorbance at 488 nm was measured using a microplate reader (Multiskan GO Microplate Spectrophotometer, Thermo Fisher Scientific, Waltham, MA, USA).

#### Wound Healing Migration Assay

Cell migration was evaluated with a wound healing assay. About 1 × 10^6^ Lovo cells were seeded into 6-well plates prepared with five straight lines on the back and allowed to grow to 100% confluency in 10% fetal bovine serum (FBS) medium. Then, a clear cell-free line was manually created by scratching a single layer using a sterile 10 μL pipette tip in each well. After scratching, the wounded monolayers were washed three times with PBS and photographed using an inverted microscope (TS100, Nikon Corp., Tokyo, Japan) at 100 × magnification as the picture for 0 h. Then, the cells were exposed to DOME (0, 250, 500, and 1,000 μg/mL) in a 2.5% FBS medium for another 24 and 48 h, and photographed at 24 and 48 h under the microscope. The width of the scratch was measured by ImageJ software (National Institutes of Health, Bethesda, MD, USA) in 10 fields. The relative migration rate was calculated as follows:

#### Transwell Migration and Invasion Assay

Migration and invasion were performed using 24-well chambers with 8 μm pore size. For the migration assay, Lovo cells were resuspended in 100 μL FBS-free medium (5 × 10^4^ cells). Then, the cells were exposed to DOME (0, 250, 500, and 1,000 μg/ml) and added to the top chamber. After that, 800 μL medium supplemented with 20% FBS was added to the lower chamber. After incubation at 37°C with 5% CO_2_ for 48 h, non-migrative Lovo cells on the upper chamber were carefully removed with a cotton swab. Then, the migrated Lovo cells were fixed in 4% paraformaldehyde for 20 min and stained with 0.1% crystal violet for 20 min. Migrated cell images were observed and photographed using an inverted fluorescence microscope (BX51, Olympus Optical Co. Ltd., Tokyo, Japan), at 200 × magnification. The images were quantitated using ImageJ software in six random fields.

For the invasion assay, the invasion capacity of Lovo cells was evaluated with the Matrigel matrix gel invasion assay. Then, the invasion assay was similarly performed as the migration assay, except that the upper chamber was pre-coated with 100 μL Matrigel (300 μg/ml) overnight before seeding with Lovo cells. The relative migration or invasion rate was calculated as follows:


$$\begin{array}{l} \text{Relative migration or invasion rate }\left(\text{\%}\right)=\frac{\text{Number of migration or invasion cells of treated group}}{\text{Number of migration or invasion cells of blank group}}\times 100\text{\%} \end{array}$$

#### TUNEL Staining Assay

Apoptotic DNA fragmentation was examined using the One Step TUNEL Apoptosis Assay kit in accordance with the manufacturer's instructions. In brief, the Lovo cells were plated in the cell culture dish (35 mm) at a density of 1 × 10^5^ cells/well. After culturing at 37°C in an incubator with 5% CO_2_ for 24 h, the cells were treated with the DOME (0, 250, 500, and 1,000 μg/mL) in triplicate for another 48 h. After treatment, the Lovo cells were washed with PBS three times, fixed with 4% paraformaldehyde for 30 min, and permeabilized with Enhanced Immunostaining Permeabilization Solution for 5 min. The cells were then washed with PBS and incubated with TUNEL assay in the dark for 1 h at 37°C. The cells were also stained with DAPI for 10 min after a rinse with PBS to visualize cell nuclei. Cyanine 3 (Cy3)-labeled TUNEL-positive cells were imaged at × 200 magnification using a confocal laser scanning fluorescence microscope (Nikon A1 confocal, Nikon, Tokyo, Japan) with 550 nm excitation and 570 nm emission wavelengths. The cells with red fluorescence were defined as apoptotic cells.

## Results

### Thirty Four Potential Active Components Were Focused on by Network Pharmacology Analysis

**Figure 2:**
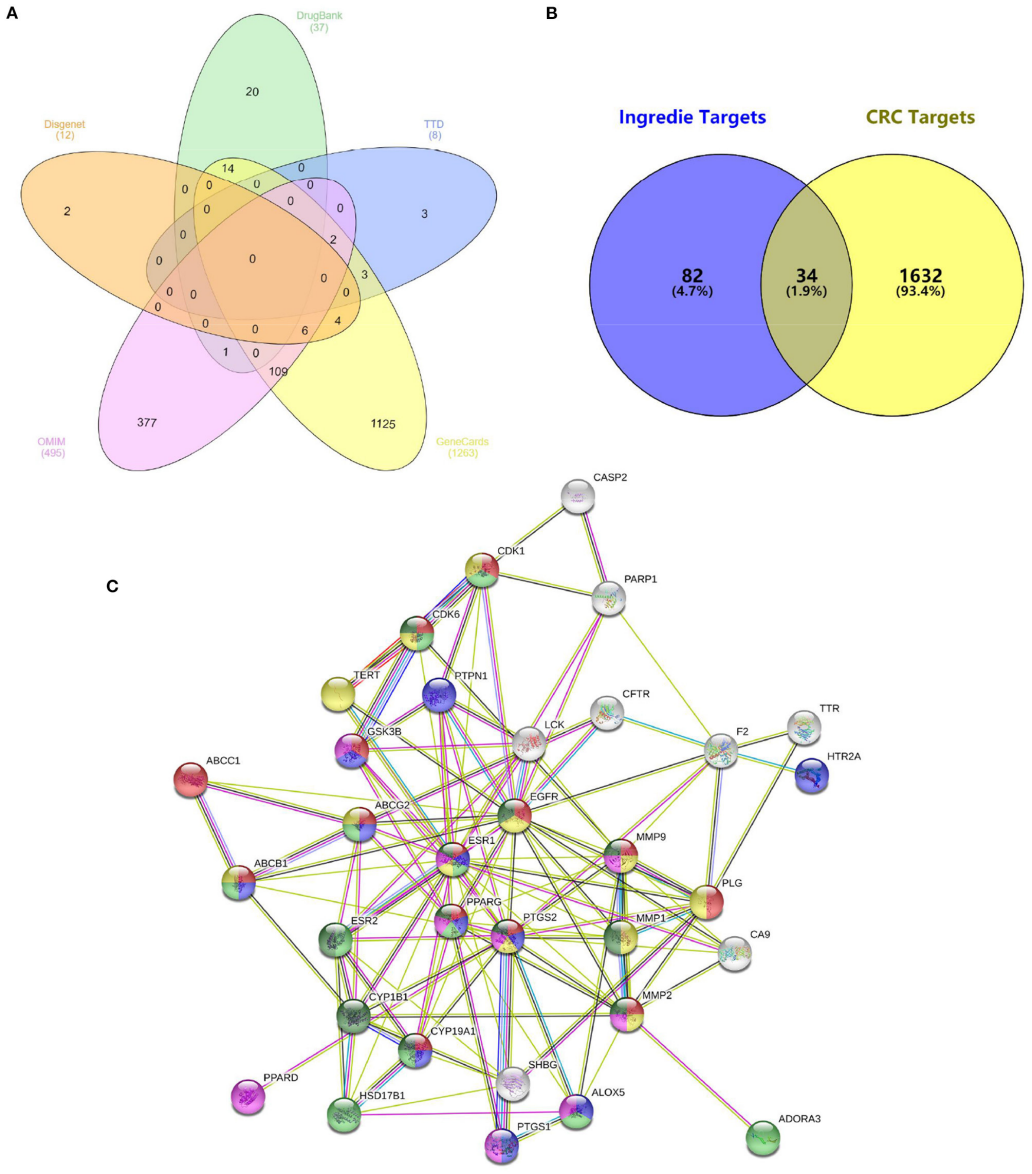
**(A)** 1,666 colon cancer-related targets. **(B)** Overlapping targets of active component targets and CRC targets. **(C)** Protein–protein interaction (PPI) network analysis.

For comprehensive identification of *D. officinale* chemical constituents, 70% aqueous methanol of *D. officinale* was analyzed using UPLC-ESI-MS/MS both in positive and negative ion scanning modes. The corresponding total ion current diagrams are shown in Supplementary Figures 1A,B. In total, 304 *D. officinale* constituents were finally identified (Table 1). According to the literature, additional 92 active components were collected (Supplementary Table 1). The in-house library totally acquired 396 candidate active components. After screening by SwissADME online tools, we explored the drug targets for the aforementioned candidate active components in *D. officinale* using SwissTargetPrediction databases, which resulted in a total of 116 putative targets (Supplementary Table 2). At the same time, 1,666 colon cancer-related targets were gathered after removing overlapping targets among different databases with 12 targets from the DisGeNet database, 495 targets from the OMIM database, 1,263 targets from the GeneCards database, 37 targets from the DrugBank database, and eight targets from the TTD database (Figure 2A).

Then, we crossed 116 *D. officinale* active component targets with 1,666 CRC-related targets using VENNY 2.1 software (Figure 2B). As a result, 34 common targets were focused on, namely, CA9, CYP1B1, ABCC1, PLG, CYP19A1, HSD17B1, SHBG, ESR1, ESR2, EGFR, FLT3, CDK1, PTGS2, CDK6, GSK3B, TTR, CFTR, ABCG2, ALOX5, PARP1, ABCB1, PPARG, PPARD, PTGS1, TERT, ADORA3, HTR2A, LCK, CASP2, MMP9, MMP1, MMP2, PTPN1, and F2. 36 active components (Supplementary Table 3). These 34 targets were linked by a network pharmacology approach.

### Construction and Analysis of Target PPI Network

The 34 common targets were submitted to STRING version 11.0 for PPI network construction (Figure 2C). As shown in Figure 2C, the results included a total of 34 nodes and 119 edges, the nodes that represent the target proteins and the edges that represent the interactions between the proteins. In this network interaction, the larger the degree is, the stronger the interaction relationship between the targets will be. It also indicates that this key target protein plays a pivotal role in regulating the network as the hub target (34). Then, Cytoscape 3.7.2 software was used for visualization and calculation of the topological parameters, such as degree, BC, and CC. Moreover, the top six targets were obtained using the value of degree as the condition for screening core targets, mainly involving ESR1, EGFR, PTGS2, MMP9, MMP2, and PPARG.

### GO and KEGG Analysis

**Figure 3:**
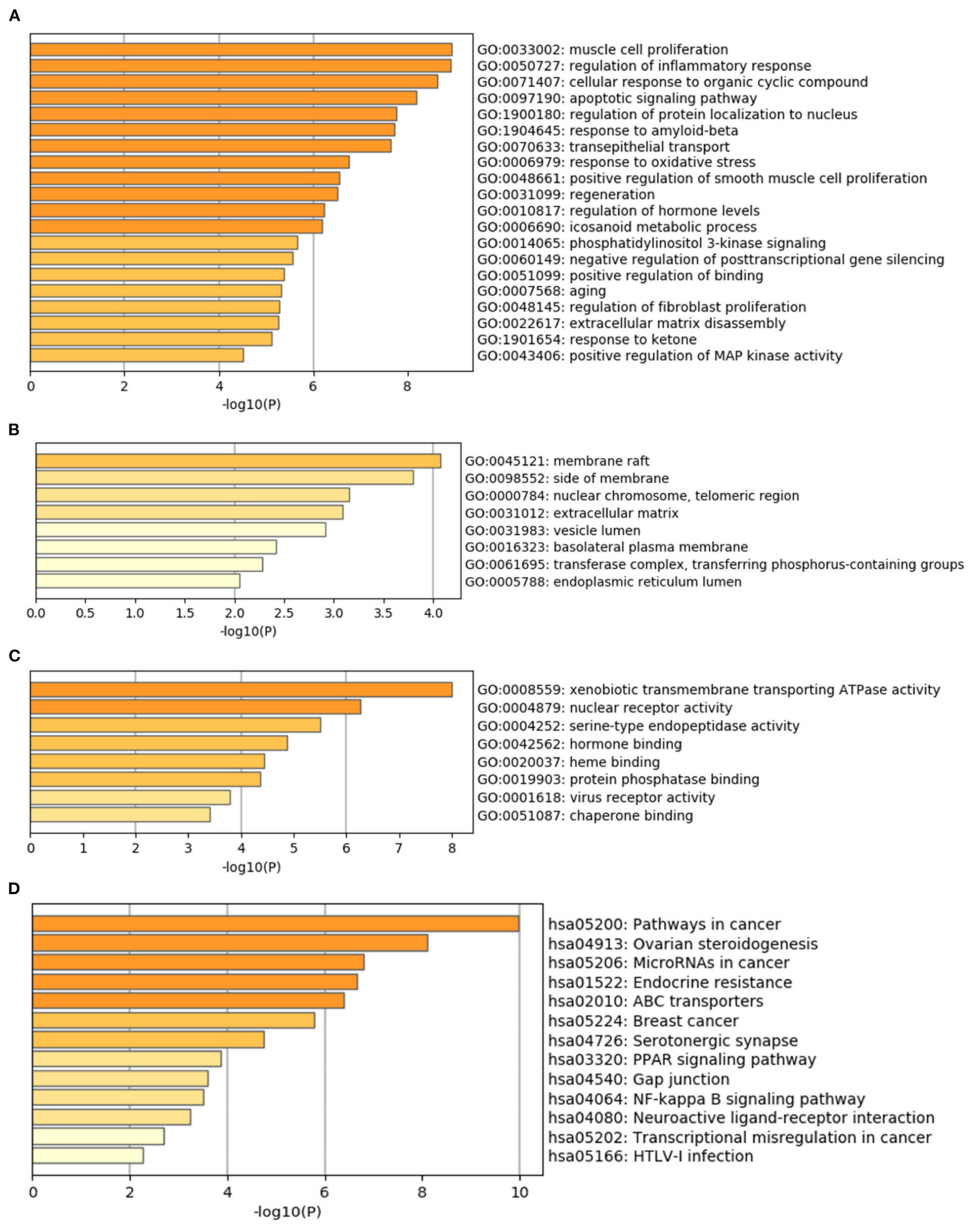
GO and KEGG pathway enrichment analyses of 34 common target genes. **(A)** Biological process analysis. **(B)** Cellular component analysis. **(C)** Molecular function analysis. **(D)** KEGG pathway analysis.

The Metascape database was used to carry out the GO enrichment analysis related to biological processes (BP), cellular components (CC), and molecular functions (MF) and KEGG pathway analysis of 34 common target genes. In the BP group, the most enriched genes concerned “muscle cell proliferation (GO:0033002),” “regulation of inflammatory response (GO:0050727),” “cellular response to organic cyclic compound (GO:0071407),” “apoptotic signaling pathway (GO:0097190),” and “regulation of protein localization to nucleus (GO:1900180)” (Figure 3A). The most enriched genes in the CC group were in “membrane raft (GO:0045121),” “side of membrane (GO:0098552),” and “nuclear chromosome, telomeric region (GO:0000784)” (Figure 3B). The genes in the MF group that were most enriched comprised “xenobiotic transmembrane transporting ATPase activity (GO:0008559),” “nuclear receptor activity (GO:0004879),” and “serine-type endopeptidase activity (GO:0004252)” (Figure 3C). KEGG pathway analysis indicated that “pathways in cancer (hsa05200),” “Ovarian steroidogenesis (hsa04913),” and “MicroRNAs in cancer (hsa05206)” were involved in the regulation of common target genes (Figure 3D). Details are listed in Supplementary Table 4. Based on these results, we were interested in and focused on the apoptotic signaling pathway, which was highly enriched in the GO and KEGG databases.

### Analyses of the Component–Target–Pathway Network

According to the predicted results of the identified active components, key targets, and top 22 related pathways using the KEGG database (Supplementary Table 4), an integrated component–target–pathway network was constructed using Cytoscape.

**Figure 4:**
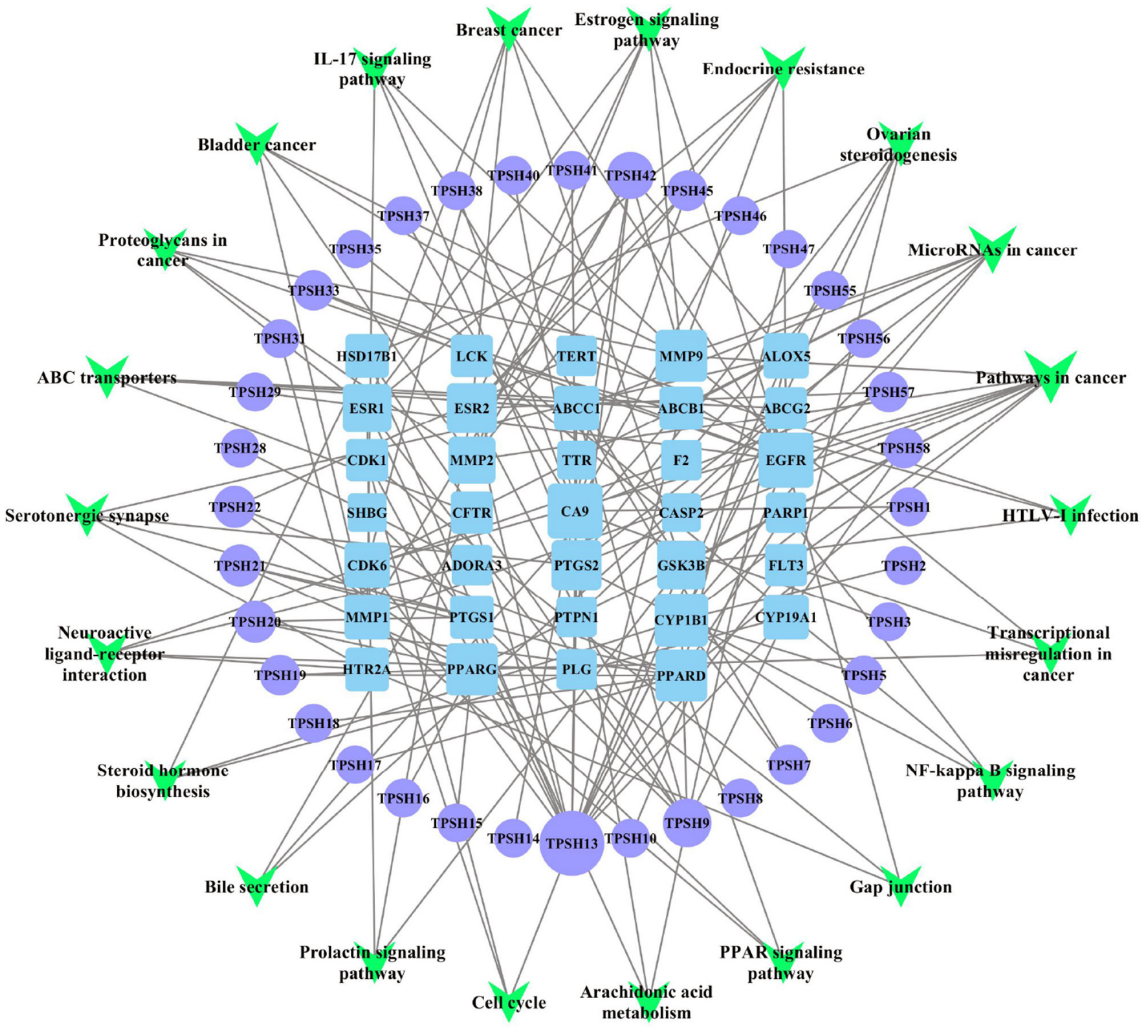
Component–target–pathway (C-T-P) network of *D. officinale* for CRC. The purple circles represent the active components of *D. officinale*, the blue squares represent the common targets, and the green inverted triangles represent the main pathways. Node size is proportional to its degree. The larger the area is, the more important it is in the network.

As shown in Figure 4, 36 core active components were obtained, which were gallic acid, isorhamnetin, protocatechuic acid, homoeriodictyol, isosakuranetin (7'-methylnaringenin), tamarixetin, Di-O-methylquercetin, naringenin, naringenin chalcone, apigenin, scopoletin (7-hydroxy-5-methoxycoumarin), 13-hydroxy-9,11-octadecadienoic acid, 9-hydroxy-10,12-octadecadienoic acid, myristic acid, pentadecanoic acid, palmitoleic acid, γ-linolenic acid, α-linolenic acid, cis-10-heptadecenoic acid, L-glutamic acid, (-)-3-(3,4-dihydroxyphenyl)-2-methylalanine, 5-hydroxy-L-tryptophan, 3,4-dihydroxy-DL-phenylalanine, spermine, ferulic acid, syringic acid, trans-4-hydroxycinnamic acid methyl ester, trans-ferulic acid, caffeic acid, p-coumaric acid, gentisic acid, hexadecanoic acid, benzamidine, rutundic acid, p-hydroxy cinnamic acid, and 3',5, 5',7-tetrahydroxyflavanone (Supplementary Table 3). Apigenin, naringenin, caffeic acid, γ-linolenic acid, α-linolenic acid, cis-10-heptadecenoic acid, tamarixetin, palmitoleic acid, 3,4-dihydroxy-DL-phenylalanine, and p-coumaric acid were found to be the top 10 important active components in this network (Supplementary Table 5). Apigenin was the most important active component (degree = 17, BC = 0.6359, and CC = 0.4607), followed by naringenin (7, 0.3811, 0.3388). In addition, ESR1, EGFR, PTGS2, MMP9, MMP2, PPARG, PLG, CYP1B1, ESR2, and CYP19A1 were found to be the top 10 important targets in this network (Supplementary Table 6).

### Molecular Docking

Molecular docking of the key active components of *D. officinale* (apigenin, naringenin, caffeic acid, γ-linolenic acid, α-linolenic acid, and cis-10-heptadecenoic acid) with the key target proteins (ESR1, EGFR, PTGS2, MMP9, MMP2, and PPARG) was performed by AutoDock 4.0.

**Figure 5:**
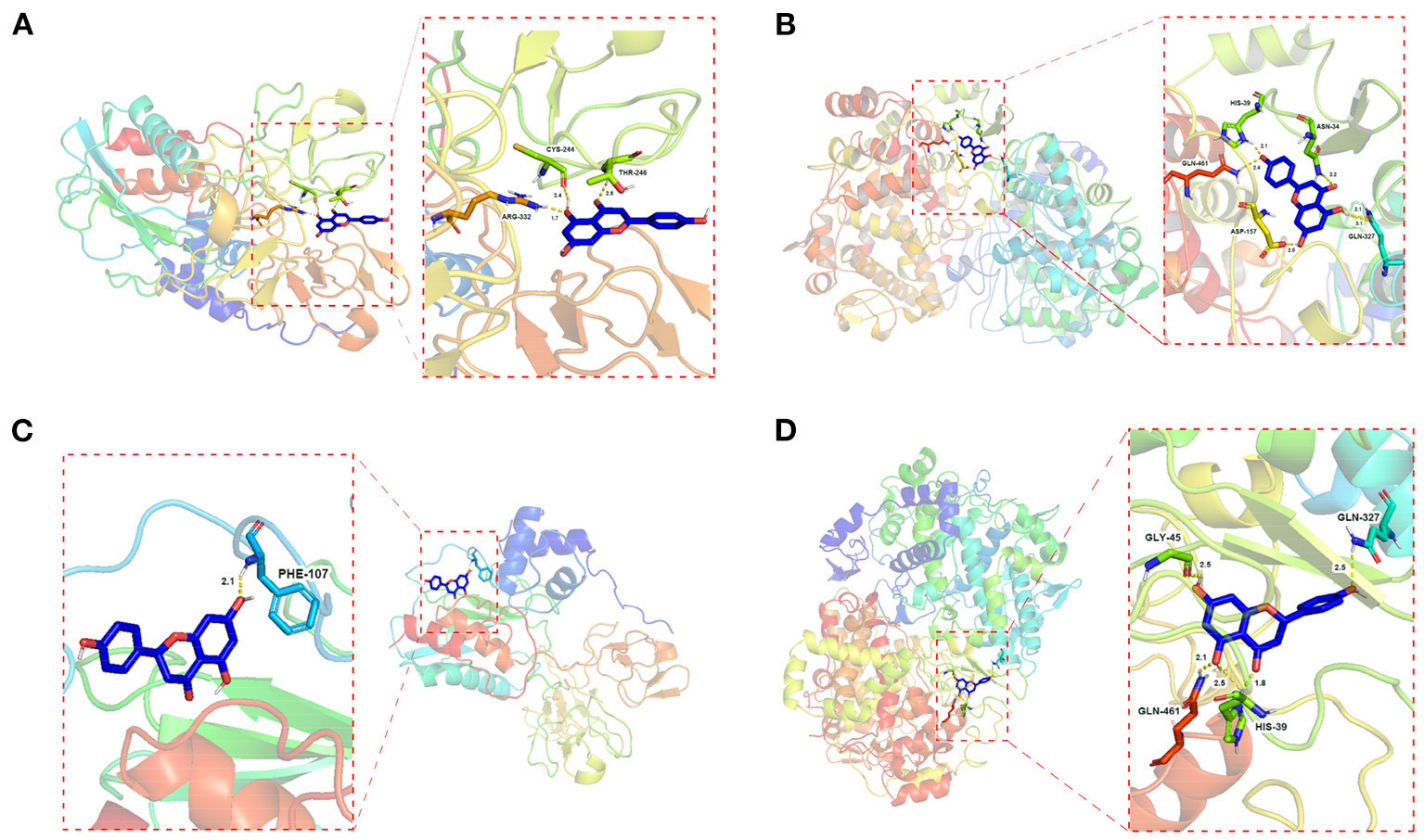
Schematic (3D) representation of the molecular model of the active component combined with the target proteins. Components: apigenin. Target proteins: **(A)** MMP9; **(B)** PTGS2; components: naringenin. Target proteins: **(C)** MMP9; **(D)** PTGS2.

The results showed that apigenin and naringenin are well-docked with PTGS2 and MMP9 and have a good affinity, suggesting that *D. officinale* has high accuracy in the treatment of CRC (Supplementary Table 7). Moreover, compared with other targets, the docking energy of apigenin with PTGS2 (−6.31 kJ/mol) was the lowest, indicating that the binding ability and stability of apigenin–PTGS2 complex were higher than those of the other targets. Apigenin was able to produce interaction with five amino acids within the binding pouch of PTGS2 such as ASN-34, GLN-327, ASP-157, GLN-461, and HIS-39 at bond lengths of 2.2, 2.1, 3.1, 2.0, 2.4, and 2.14 Å, respectively. The docking model is shown in Figure 5B. In addition, apigenin was docked with MMP9, and the binding energy was −5.20 kJ/mol. Also, apigenin was able to produce interaction with three amino acids within the binding pouch of MMP9 such as THR-246, ARG-332, and CYS-244 at bond lengths of 2.5, 1.7, and 2.4 Å, respectively. The docking model is shown in Figure 5A. The docking binding energies of naringenin with PTGS2 and MMP9 were −6.20 and −5.81 kJ/mol, respectively. Naringenin was able to produce interaction with four amino acids within the binding pouch of PTGS2 such as HIS-39, GLN-461, GLY-45, and GLN-327 at bond lengths of 1.8, 2.5, 2.1, 2.5, and 2.5 Å, respectively. Also, naringenin was able to produce interaction with one amino acid (PHE-107) within the binding pouch of MMP9 at a bond length of 2.1 Å. The docking model is shown in Figures 5C,D.

### Experimental Evaluation

#### MTT Assay

**Figure 6:**
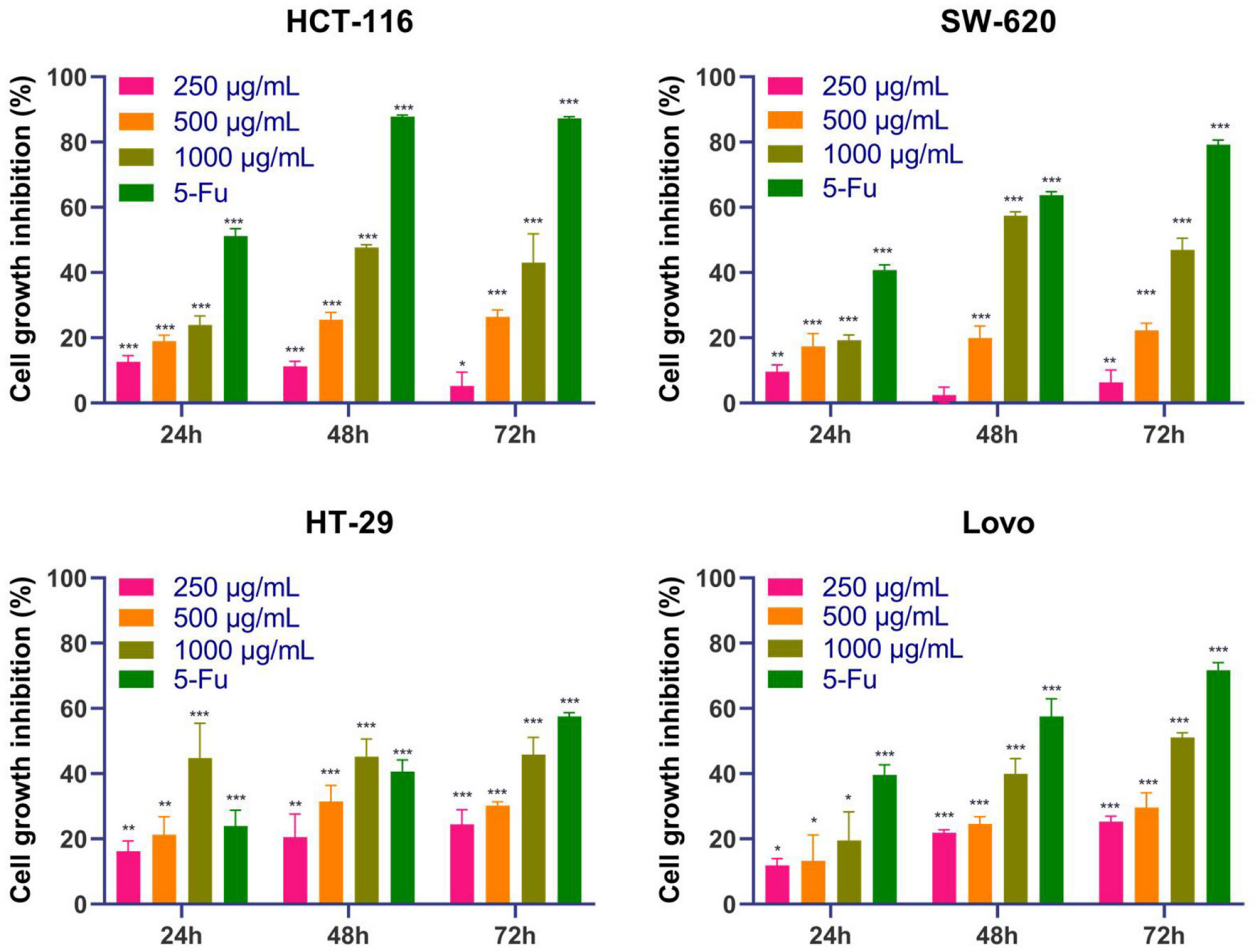
Effects of DOME on the proliferation of CRC cell lines. The cell viability of HT-29, Lovo, SW-620, and HCT-116 cells treated with DOME for 24, 48, and 72 h were determined by MTT assay. The error bars represent SEM (*P < 0.05, **P < 0.01, ***P < 0.001, when compared with blank group).

To investigate the antitumor effect of the DOME on CRC, the treated HT-29, Lovo, SW-620, and HCT-116 cells were analyzed using an MTT assay. As shown in Figure 6, the results showed that the DOME decreased the proliferation of the four CRC cell lines in a dose- and time-dependent manner. In addition, the inhibitory rate was 1,000 μg/ml DOME ≤ 20% on the four CRC cells at 24 h, except the DOME on HT-29 cells (44.83%). However, the inhibitory rate was 1,000 μg/ml DOME ≥40% on these CRC cells at 48 h, which is close to the effect at 72 h. Therefore, this study chose 48 h as the time of the DOME on CRC cells in the subsequent experiments.

#### Wound Healing Migration Assay and Transwell Migration and Invasion Assay

**Figure 7:**
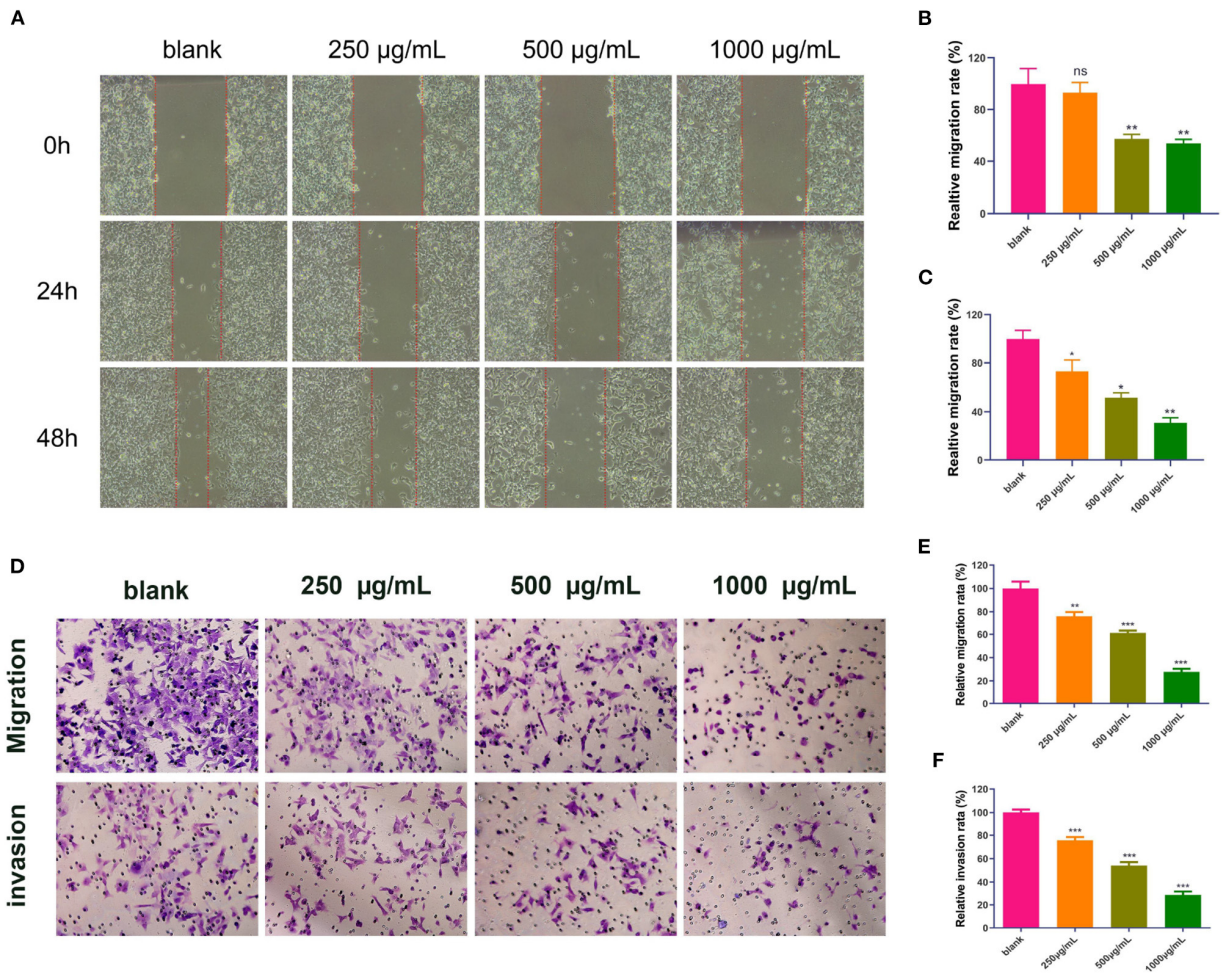
DOME suppresses migration and invasion of Lovo cells. **(A)** Images of wound healing assay; quantification of wound healing assay in Lovo cells at 24 h **(B)** and 48 h **(C)**; **(D)** transwell assay in Lovo cells. **(E)** quantification of transwell migration assay in Lovo cells; **(F)** quantification of transwell invasion assay in Lovo cells. The error bars represent SEM (*P < *0.05*, **P < *0.01*, ***P < *0.001*, when compared with the blank group).

Because MMP-2 and MMP-9 are important executors in cell migration and invasion, and based on the previous results in the component–target–pathway network analysis and molecular docking analysis, we further assessed the inhibitory effects of the DOME on the *in vitro* migration and invasion potential of Lovo cells using the wound healing migration assay and transwell assay, respectively. These results showed that the DOME significantly inhibited Lovo cell migration in a dose- and time-dependent manner (Figures 7A–C). To further assess the effects of the DOME on the migration of Lovo cells *in vitro*, the transwell migration assay was employed. The transwell migration assay also revealed that the DOME markedly weakened the migration ability of Lovo cells (*P* < 0.01, Figures 7D–F), further confirming the result of the wound healing assay.

Furthermore, cell invasion ability was determined by a transwell invasion assay. Compared to the blank group, the number of invaded cells in the DOME group was significantly reduced in a dose-dependent manner (Figures 7E,F). These results suggested that the DOME could suppress Lovo cell migration and invasion *in vitro*.

#### TUNEL Staining

**Figure 8:**
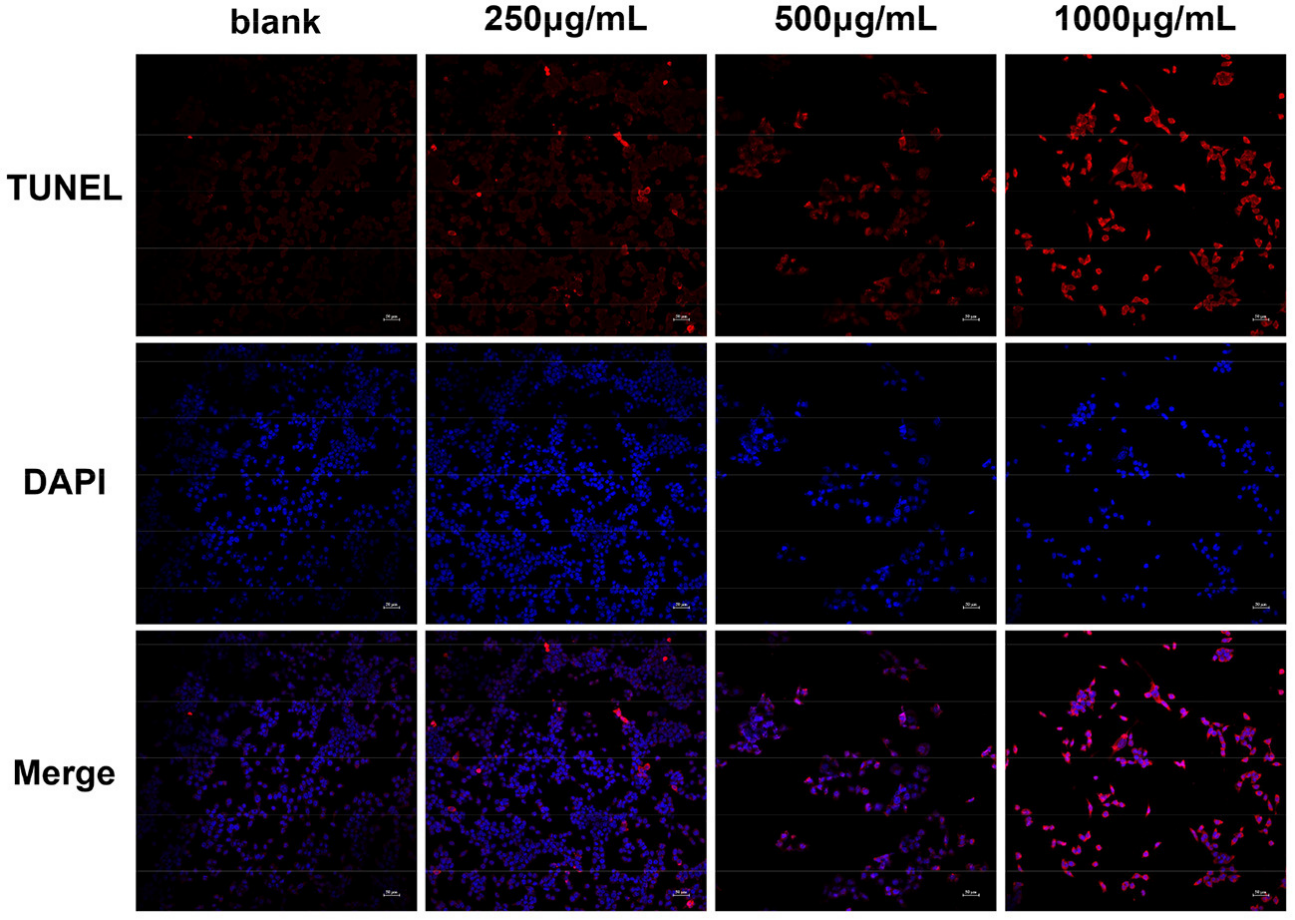
Effect of DOME on cell apoptosis in Lovo cells. The cell apoptosis was determined using TUNEL assay, TUNEL-positive nuclei in red fluorescent color, and total nuclei staining with DAPI. Scale bar: 50 μm.

According to the predicted results of the GO and KEGG pathway enrichment analyses and to further confirm the apoptosis-inducing activities of DOME in Lovo cells, TUNEL staining was also performed. The cell apoptosis in Lovo cells was determined with TUNEL assay after DOME treatments for 48 h. As shown in Figure 8, TUNEL-positive nuclei (representing apoptotic cells) were stained with red fluorescence, while total nuclei stained with DAPI and exhibited blue fluorescence. Figure 8 shows that the DOME remarkably increased TUNEL-positive (red) cell number. The result demonstrated that the DOME induces apoptosis in Lovo cells, and this effect was significantly enhanced in a dose-dependent manner.

## Discussion

Currently, chemotherapy is the conventional treatment method for CRC, such as 5-Fu. However, chemotherapy has many adverse effects (35). A number of prior studies have shown that *D. officinale* exhibits excellent bioactivity against CRC (9, 10). Used as a medicine and food dual-use product for more than a thousand years, *D. officinale* has fewer side effects (1). However, the underlying mechanisms of anti-colon cancer effects of *D. officinale* remain unclear because of the complex compositions. The core active components and core target of *D. officinale*, which play the key role against the CRC, are unclear. We applied metabolomics combined with network pharmacology to screen the active components, drug targets, and singling pathway. Moreover, we performed further experimental validation using a series of cellular functional and molecular biological assays *in vitro*. Overall, our results did not only clarify the anti-CRC effective components and their potential targets of *D. officinale* but also offer in-depth knowledge of the molecular mechanisms of *D. officinale*.

In this study, 304 metabolites of active components in *D. officinale* 70% methanol extracts were determined by UPLC-ESI-MS/MS metabonomic analysis, while 178 compounds were identified by UPLC-MS/MS from the *D. officinale* anhydrous ethanol extracts as reported in the literature (36). It may be due to the different origins of *D. officinale* or the different extraction solvents used. Furthermore, in order to fully establish the component library of *D. officinale* and make the network pharmacological analysis more accurate (37), we used the literature mining method (PubMed, CNKI.net, Cqvip.com, and Web of Science) to search for those that had been reported as the key active components of *D. officinale*, while had not been identified in UPLC-ESI-MS/MS analysis. Overall, 92 active components of *D. officinale* were obtained. So, the in-house library totally acquired 396 active components. Subsequently, 116 putative targets were obtained by screening using the SwissADME online tools and SwissTargetPrediction databases. Then, 1,666 colon cancer-related targets were gathered from the DisGeNet database, OMIM database, GeneCards database, DrugBank database, and the TTD database (Figure 2A); 34 common targets were acquired after crossing 116 *D. officinale* active component targets with 1,666 CRC-related targets using VENNY 2.1 software.

Network pharmacology analysis results showed that the key active components mainly involved apigenin, naringenin, caffeic acid, γ-linolenic acid, α-linolenic acid, and cis-10-heptadecenoic acid, and the top six targets mainly involved ESR1, EGFR, PTGS2, MMP9, MMP2, and PPARG. These results involving key active components naringenin and caffeic acid were consistent with the literature report (36). The results of molecular docking suggested that the aforementioned key molecules are well-docked with important target proteins and have a good affinity, suggesting that *D. officinale* has high accuracy in the treatment of CRC (Supplementary Table 7). Moreover, the docking energy of apigenin with PTGS2 (−6.31 kJ/mol) was the lowest, followed by naringenin with PTGS2 (−6.20 kJ/mol), naringenin with MMP9 (−5.81 kJ/mol), and apigenin with MMP9 (−5.20 kJ/mol) (Figure 5), indicating that apigenin and naringenin in *D. officinale* were the most important active components, and PTGS2 and MMP9 were the most important targets that play the role of against CRC.

The finding that apigenin and naringenin were the most important active components is consistent with previous studies which have demonstrated that apigenin and naringenin have inhibitory effects against CRC (38–41). However, it is worth noting that the content of naringenin in *D. officinale* is much more than apigenin, consistent with a previous study (42). The study showed that the content of naringenin in *D. officinale* was generally higher, and the average mass fraction of the components reached 26.87 μg·g^−1^. Correspondingly, the component mass fraction of apigenin in *D. officinale* is ≤ 0.5 μg·g^−1^, it mainly exists in the form of flavonoid carbon glycosides, with apigenin as the core, rather than its free state (43–45). Therefore, naringenin might play a more important role than apigenin. However, there is also a possibility that the flavonoid carbon glycosides with apigenin as the nucleus degrade into free-state apigenin to exert anti-CRC effects.

MMP-2 and MMP-9 are important executors in cell migration and invasion (46, 47), and PTGS2 has been recognized to play an important role in regulating cell migration, invasion, and proliferation (48). In addition, in accordance with the results of molecular docking, a previous study has demonstrated that apigenin and naringenin can inhibit the invasion and migration of colon cancer cells by inhibiting the epithelial-to-mesenchymal transition (EMT) of CRC (49, 50). Hence, it could conceivably be hypothesized that the mechanism of *D. officinale* against CRC is closely related to the inhibition of tumor invasion and migration. The experiments of wound healing migration assay and transwell migration and invasion assay in this study further verified the conjecture. Despite these promising results, there were also some limitations to this research. We have not proven that apigenin and naringenin could exert anti-colon cancer effects *via* regulating PTGS2 and MMP9 with additional experiments in this study. The experimental evaluation was conducted only *in vitro*. Further *in vivo* animal experiments still need to be performed to confirm and validate these findings.

The GO and KEGG analysis results showed that the target proteins involved in the main pathways were “muscle cell proliferation (GO:0033002),” “regulation of inflammatory response (GO:0050727),” “cellular response to organic cyclic compound (GO:0071407),” “apoptotic signaling pathway (GO:0097190),” “regulation of protein localization to nucleus (GO:1900180),” “pathways in cancer” (hsa05200), “ovarian steroidogenesis” (has04913), and “microRNAs in cancer” (hsa05206) (Figures 3A,D). As shown in Supplementary Figure 2, the cancer-related KEGG pathways were determined as pathways related to the “pathways in cancer”: PPAR signaling pathway, MAPK signaling pathway, calcium signaling pathway, cAMP signaling pathway, cytokine–cytokine receptor interaction, HIF-1 signaling pathway, cell cycle, p53 signaling pathway, mTOR signaling pathway, PI3K-Akt signaling pathway, apoptosis, Wnt signaling pathway, Notch signaling pathway, Hedgehog signaling pathway, TGF-beta signaling pathway, VEGF signaling pathway, focal adhesion, ECM–receptor interaction, adherens junction, JAK-STAT signaling pathway, and estrogen signaling pathway (51). Combined with the core protein targets analyzed in the previous network pharmacology (mainly ESR1, EGFR, PTGS2, MMP9, MMP2, PPARG, etc.), we speculate that the subcellular pathways that may play a role in inhibiting colon cancer proliferation, invasion, and migration are EGFR-associated signaling pathways (EGFR/Raf/MEK/ERK cascades) and PPAR signaling pathway. As shown in Figure 4D, “PPAR signaling pathway (hsa03320)” was also the eighth most enriched pathway in the KEGG pathway analysis. As shown in Supplementary Figures 3, 4, combined with the protein targets screened in the previous network pharmacology, we suppose miR-145 suppresses tumorigenesis and metastasis of the colorectal cancer cells by inhibiting the expression of EGFR. Dysregulation of specific miRNAs has been associated with certain types of cancer, where they may act as either oncogenes or tumor suppressors, depending on their target genes (52). For example, *in situ* hybridization detected accumulation of miR-145 in normal colon epithelia with no signal from adenocarcinomas cells. Loss of miR-145 in various tumors suggests its role as a tumor suppressor. miR-145 is downregulated in colorectal cancer (53, 54). In fact, miR-145 has been well-documented as a tumor suppressor gene in multiple tumor types because of its anti-proliferative and pro-apoptotic effects. Several reports suggest that miR-145 is a suppressor of metastasis. For example, miR-145 negatively regulates MUC1 and suppresses invasion and metastasis of breast cancer cells (55), and miR-145 suppresses proliferation, metastasis, and EMT of colorectal cancer *via* suppressing the EGFR-associated signaling pathway (56–58). In this study, other mechanisms in the aforementioned signal pathways might also play an important role. Further research is required to be performed to better understand the underlying mechanisms.

## Conclusion

In conclusion, the present results demonstrated that the core active components are apigenin and naringenin, and the core targets are PTGS2 and MMP-9 when *D. officinale* exerts antitumor effects on CRC through a combination of network pharmacology, metabolomics, and molecular docking. Moreover, the apoptotic signaling pathway and inhibition of tumor invasion and migration signaling pathway had been validated to play a vital role in *D. officinale* against CRC. Our findings provided valuable insights into exploring the mechanism of action of *D. officinale* against CRC.

## Data Availability Statement

The datasets presented in this study can be found in online repositories. The names of the repository/repositories and accession number(s) can be found at: MetaboLights; MTBLS4678.

## Author Contributions

ST: conceptualization, data curation, writing—original draft preparation, writing—review and editing, and visualization. ST, JL, and HW: methodology and validation. ST, RH, and ZR: software. ST and HW: formal analysis. ST, SD, and WH: investigation. GW: resources, supervision, project administration, and funding acquisition. All authors contributed to the article and approved the submitted version.

## Funding

This work was supported by the Innovation project of Dendrobium truth-seeking in Guangzhou University of Traditional Chinese Medicine (No. 2019shqz0209801), the 2018 Shaoguan City Science and Technology Plan Project: Special Project of Industry-University-Research Cooperation (No. 2018CS11919), and the 2019 Guangdong Province Special Fund for Science and Technology (Big project + task list) Project: Ecological Cultivation and Sustainable Utilization of Danxia *Dendrobium officinale*, a rare Southern Medicine in Guangdong Province (Nos. 2019gdskjzxzj-zt3-2 and 210316166270419).

## Conflict of Interest

The authors declare that the research was conducted in the absence of any commercial or financial relationships that could be construed as a potential conflict of interest.

## Publisher's Note

All claims expressed in this article are solely those of the authors and do not necessarily represent those of their affiliated organizations, or those of the publisher, the editors and the reviewers. Any product that may be evaluated in this article, or claim that may be made by its manufacturer, is not guaranteed or endorsed by the publisher.

## Abbreviations

ADME: absorption, distribution, metabolism, and excretion
BC: betweenness centrality
CC: closeness centrality
CRC: colorectal cancer
*D. officinale*: *Dendrobium officinale*
DOME: *D. officinale* methanolic extract
DMSO: dimethyl sulfoxide
FBS: fetal bovine serum
GO: Gene Ontology
5-Fu: 5-fluorouracil
C-T-P network: component–target–pathway network
KEGG: Kyoto Encyclopedia of Genes and Genomes
MF: molecular functions
MTT, 3-[4, 5-dimethylthiazol-2-yl]-2: 5-diphenyltetrazolium bromide
OMIM: Online Mendelian Inheritance in Man
PDB: Protein Data Bank
PPI: protein–protein interaction
STRING: Search Tool for the Retrieval of Interacting Genes/Proteins
TTD: Therapeutic Target Database.
