# Supplementary material for: Anti-colon Cancer Effects of Dendrobium officinale Kimura & Migo Revealed by Network Pharmacology Integrated With Molecular Docking and Metabolomics Studies
Source: Front Med (Lausanne). 2022 Jun 30;9:879986. doi: 10.3389/fmed.2022.879986 (PMC9280342; doi:10.3389/fmed.2022.879986)
Supplement: Supplementary file 1 [file Data_Sheet_1.docx]

**Supporting Information**

Table S1. The chemical composition of *D. officinale* supplemented from literature

Table S2. 116putative targets

Table S3. 36 components were linked with these 34 targets

Table S4. KEGG pathway enrichment

Table S5. The top ten important components

Table S6. The top ten important targets

Table S7. The binding energy values of the core active components and Target Proteins

Figure S1. Total ion flow chart of 70% aqueous methanol of *D. officinale*. (A) ESI (–); (B) ESI (+).

Figure S2. pathways in cancer

Figure S3. MirRNA in Cancer

Figure S4. An overview of WNT, EGFR, TP53 and TGFb signaling pathways in CRC and the regulation of their key molecule by miRNAs.

**Table S1. The chemical composition of *D. officinale* supplemented from literature**

| **ID** | **Compound** | **Ref.** | **Molecular Formula** | **Mw (g/mol)** |
| --- | --- | --- | --- | --- |
| **Stilbene** | | |  |  |
| 1 | Dendrocandin A | [1] | C_17_H_20_O_5_ | 304.1308 |
| 2 | Dendrocandin B | [1] | C_27_H_30_O_8_ | 482.1951 |
| 3 | Dendrocandin C | [2] | C_16_H_18_O_5_ | 289.1086 |
| 4 | Dendrocandin D | [2] | C_17_H_19_O_5_ | 303.1232 |
| 5 | Dendrocandin E | [2] | C_15_H_16_O_5_ | 275.0912 |
| 6 | Dendrocandin F | [3] | C_32_H_32_O_8_ | 543.2026 |
| 7 | Dendrocandin G | [3] | C_31_H_30_O_8_ | 529.1866 |
| 8 | Dendrocandin H | [3] | C_30_H_22_O_9_ | 525.1190 |
| 9 | Dendrocandin I | [3] | C_32_H_32_O_8_ | 543.2026 |
| 10 | Dendrocandin J | [4] | C_31_H_29_O_8_ | 529.1880 |
| 11 | Dendrocandin K | [4] | C_30_H_27_O_8_ | 515.1729 |
| 12 | Dendrocandin L | [4] | C_30_H_21_O_8_ | 509.1236 |
| 13 | Dendrocandin M | [4] | C_26_H_29_O_8_ | 469.1870 |
| 14 | Dendrocandin N | [4] | C_25_H_25_O_7_ | 437.1604 |
| 15 | Dendrocandin O | [4] | C_25_H_25_O_8_ | 453.1556 |
| 16 | Dendrocandin P | [4] | C_30_H_28_O_8_ | 515.1706 |
| 17 | Dendrocandin Q | [4] | C_30_H_27_O_8_ | 515.1708 |
| 18 | Dendrocandin R | [4] | C_19_H_22_O_5_ | 329.1389 |
| 19 | 4，4'-dihydroxy-3,5-dimethoxybibenzyl | [1] | C_16_H_18_O_4_ | 274 |
| 20 | 3，4-dihydroxy-5,4‘-dimethoxybibenzyl | [1] | C_16_H_18_O_4_ | 274 |
| 21 | 3-O-methylgigantol | [1] | C_17_H_20_O_4_ | 288 |
| 22 | dendrophenol | [1] | C_17_H_20_O_5_ | 304 |
| 23 | gigantol | [1] | C_16_H_18_O_4_ | 274 |
| 24 | erianin | [5] | C_18_H_22_O_5_ | 318.364 |
| 25 | 3，4'-dihydroxy-5-methoxybibenzyl | [6] | C_15_H_16_O_3_ | 244 |
| 26 | dihydroresveratrol | [6] | C_14_H_14_O_3_ | 230 |
| 27 | dendromoniliside E | [6] | C_28_H_38_O_14_ | 598 |
| 28 | denbinobin | [6] | C_16_H_12_O_5_ | 284 |
| 29 | 2，4，7-trihydroxy-9，10-dihydrophenanthrene | [6] | C_14_H_12_O_3_ | 228 |
| 30 | 4，5-dihydroxy-3，3'-dimethoxybibenzyl | [7] | C_16_H_18_O_4_ | 274 |
| 31 | 2，3，4，7-tetramethoxyphenanthrene | [8] | C_18_H_18_O_4_ | 298 |
| 32 | Chrysotoxene | [9] | C_18_H_18_O_5_ | 314 |
| 33 | erianthridin | [10] | C_16_H_16_O_4_ | 272 |
| 34 | ephemeranthol A | [10] | C_16_H_16_O_4_ | 272 |
| 35 | 2, 7-dihydroxy-3, 4-dimethoxyphenanthrene | [10] | C_16_H_14_O_4_ | 270 |
| 36 | confusarin | [10] | C_17_H_12_O_5_ | 296 |
| 37 | trigonopol B | [11] | C_25_H_26_O_7_ | 438 |
| 38 | moellenoside A | [10] | C_25_H_29_O_12_ | 521 |
| **phenols** | | |  |  |
| 39 | koaburaside | [12] | C_16_H_23_O_7_ | 327 |
| 40 | khaephuouside | [13] | C_20_H_28_O_13_ | 476 |
| 41 | leonuriside A | [13] | C_14_H_20_O_9_ | 332 |
| 42 | 2-methoxyphenyl-1-O-β-D-apiofuromosyl-( 1→2) -β-D-glucopyranoside | [13] | C_18_H_26_O_11_ | 418.147 5 |
| 43 | erigeside Ⅱ，dictamnoside A | [12] | C_17_H_24_O_8_ | 356 |
| 44 | paprazine | [12] | C_17_H_17_NO_3_ | 283.322 |
| 45 | moupinamide | [12] | C_18_H_19_NO_4_ | 313.348 |
| 46 | dihydro-feruloyltyramine | ^[24]^ | C_18_H_21_NO_4_ | 315.348 |
| 47 | p-hydroxyphenylpropionyl tyramine | ^[24]^ | C_17_H_19_NO_3_ | 285.348 |
| 48 | 4-hydroxy-3-methoxybenzyl alcohol | [7] | C_8_H_10_O_3_ | 154.163 |
| 49 | N-trans-feruloyl tyramine | [7] | C_18_H_19_NO_4_ | 313 |
| 50 | Cis-feruloyl p-hydroxyphenethylamine | [7] | C_18_H_19_NO_4_ | 313 |
| 51 | N-trans-cinnamoyltyramine | [7] | C_17_H_17_NO_2_ | 267 |
| **Littermate** | | |  |  |
| 52 | n-octacostyl ferulate | [6] | C_38_H_66_O_4_ | 586 |
| 53 | defuscin | [6] | C_40_H_75_O_2_ | 587 |
| 54 | n-triacontyl cis-p-coumarate | [6] | C_40_H_75_O_2_ | 587 |
| 55 | heptadecanoic acid | [6] | C_17_H_34_O_2_ | 270 |
| 56 | hentriacontane | [6] | C_31_H_64_O | 452 |
| **Phenylpropanoid** | | |  |  |
| 57 | p-hydroxy-phenylpropionic acid | [7,12] | C_9_H_10_O_3_ | 166.174 |
| 58 | p-hydroxy cinnamic acid | [7,12] | C_9_H_8_O_3_ | 164.158 |
| 59 | trans-3, 4, 5-trimethoxyl-cinnamyl alcoho | [10] | C_12_H_10_O_4_ | 218 |
| 60 | (1R ) -1-( 4-hydroxy-3，5-dimethoxylphenyl) propan-1-ol-4-O-β-D-glucopyranoside | [13] | C_17_H_26_O_9_ | 374 |
| 61 | ω-hydroxypropioguaiacone | [11] | C_10_H_12_O | 148 |
| **Lignins** | | |  |  |
| 62 | dhydroconiferyl dihydro-p-cumarate | [12] | C_19_H_22_O_5_ | 330 |
| 63 | (+)-syringaresinol-4-O-β-D-glucopyranoside | [13] | C_20_H_26_O_8_ | 394 |
| 64 | icariol A_2_-4-O-β-D-glucopyranoside | [13] | C_28_H_38_O_14_ | 598 |
| 65 | (+) -lyoniresinol-3a-O-β-D-glucopyranoside | [13] | C_26_H_38_O_13_ | 558 |
| 66 | (－)-secoisolariciresinol | [13] | C_20_H_27_O_6_ | 363 |
| 67 | (－)syringaresinol | [7,13] | C_22_H_26_O_8_ | 418 |
| 68 | syringaresinol-4, 4'-O-bis-β-D-glucoside | [13]］ | C_26_H_36_O_13_ | 556 |
| 69 | dihydrosyringin | [12]］ | C_17_H_26_O_9_ | 374 |
| 70 | magnolenin C | [10] | C_29_H_41_O_12_ | 581 |
| 71 | officinalioside | [10] | C_28_H_36_O_14_ | 596 |
| 72 | pinoresinol-4-O-β-D-glucopyranoside | [10] | C_26_H_32_O_11_ | 520 |
| 73 | (8S, 7'R, 8'S) -5, 5'-dimethoxy-lariciresinol | [11] | C_23_H_30_O_8_ | 434 |
| 74 | (7R, 8S, 7'R, 8'S) -syringaresinol-4-O-β-D-glucopyranoside | [11] | C_28_H_38_O_13_ | 582 |
| 75 | (7R, 8S, 7'R, 8'S) -syringaresinol | [11] | C_22_H_26_O_8_ | 418 |
| 76 | (7S, 8S, 8'R) -lyoniresinol | [11] | C_22_H_20_O_8_ | 412 |
| **Lactones** | | |  |  |
| 77 | aduncin | [6] | C_14_H_19_O_6_ | 283 |
| 78 | digiprolactone | [6] | C_11_H_15_O_3_ | 195 |
| **Flavonoids** | | |  |  |
| 79 | 3'，5，5'，7 -tetrahydroxyflavanone | [12] | C_15_H_12_O_6_ | 288 |
| 80 | cyanidin 3-[2-(glucosyl)-6-(sinapoyl) glucoside]-5-glucoside | [12] | C_44_H_51_O_25_ | 979 |
| 81 | cyanidin 3-O-rutinoside | [12] | C_27_H_31_O_16_ | 611 |
| 82 | delphinidin 3-glucoside-7,3′-di-[6-(sinapoyl)glucoside] | [12] | C_55_H_61_O_29_ | 1185 |
| 83 | cyanidin 3-[6-(sinapoyl)glucoside]-5-glucoside | [12] | C_38_H_41_O_20_ | 817 |
| 84 | cyanidin 3-[6-sinapoyl-2-O-(2-(sinapoyl)glucosyl)-glucoside]-5-glucoside | [12] | C_55_H_61_O_29_ | 1185 |
| 85 | cyanidin 3-[6-(sinapoyl)glucoside] | [12] | C_32_H_31_O_15_ | 655 |
| 86 | cyanidin-3-[6-sinapoyl-2-O-(2-(sinapoyl)glucosyl)-glucoside | [12] | C_50_H_51_O_24_ | 1053 |
| **Steroids** | | |  |  |
| 87 | β-sitosterol | [6] | C_30_H_52_O | 428 |
| 88 | stigmast-5-en-3β-ol-7-one | [11] | C_29_H_46_O_2_ | 426 |
| 89 | daucosterol | [6] | C_35_H_60_O_6_ | 576 |
| **Others** | | |  |  |
| 90 | 5-hydroxymethyl-furaldehyde | [6] | C_6_H_6_O_3_ | 126 |
| 91 | 3, 5-dimethoxyphenethylamines | [11] | C_10_H_15_NO_2_ | 181 |
| 92 | (6S, 9S) -6-hydroxy-3-oxo-α-ionol | [11] | C_13_H_20_O_3_ | 224 |

**Table S2 116putative targets**

| CA2 |  | ABCG2 |  | HTR2B |
| --- | --- | --- | --- | --- |
| CA7 |  | AKR1B10 |  | HTR1A |
| CA1 |  | TNKS2 |  | HTR2A |
| CA3 |  | TNKS |  | HTR2C |
| CA6 |  | ALOX5 |  | HTR7 |
| CA12 |  | PARP1 |  | HMGCR |
| CA14 |  | ABCB1 |  | LCK |
| CA9 |  | PPARG |  | CASP2 |
| FUT7 |  | PPARA |  | FUCA1 |
| CA4 |  | PPARD |  | MMP9 |
| CA5B |  | FFAR1 |  | MMP1 |
| CA5A |  | FABP3 |  | MMP2 |
| CA13 |  | FABP4 |  | PTPN1 |
| XDH |  | FABP5 |  | HCAR2 |
| CYP1B1 |  | FABP2 |  | EGLN1 |
| AKR1B1 |  | PTGS1 |  | INMT |
| NOX4 |  | FAAH |  | SLC6A1 |
| ABCC1 |  | TERT |  | GABRA1 |
| PLG |  | FABP1 |  | GABRA3 |
| CYP19A1 |  | SCD |  | GABRA2 |
| HSD17B1 |  | SPHK1 |  | GABBR2 |
| SHBG |  | CAPN1 |  | GABRR1 |
| CBR1 |  | GRIK1 |  | SLC6A11 |
| ESR1 |  | GRIA1 |  | SLC6A13 |
| ESR2 |  | ADORA3 |  | SIRT3 |
| CHRNA7 |  | GRIK5 |  | SIRT2 |
| EGFR |  | SLC1A1 |  | TAAR1 |
| PIM1 |  | GRM4 |  | CNR1 |
| ADORA1 |  | GRM3 |  | TRPV1 |
| ADORA2A |  | GRIA4 |  | GPR119 |
| CDK5R1 |  | GRM5 |  | F2 |
| MAOA |  | GRM8 |  | F10 |
| FLT3 |  | GRIK2 |  |  |
| CDK1 |  | GRIK3 |  |  |
| ACHE |  | GRM1 |  |  |
| PTGS2 |  | GRM7 |  |  |
| CDK6 |  | GRIA2 |  |  |
| SYK |  | GRM6 |  |  |
| GSK3B |  | GRM2 |  |  |
| TTR |  | KDM4E |  |  |
| CSNK2A1 |  | FYN |  |  |
| CFTR |  | CACNA2D1 |  |  |

**Table S3. 36 components were linked with these 34 targets**

| [ID](https://www.chemsrc.com/baike/946915.html) | Name | Gene Symble ID |
| --- | --- | --- |
| TPSH1 | Gallic acid | CA9 |
| TPSH2 | Isorhamnetin | CYP1B1 |
| TPSH3 | Protocatechuic acid | CA9 |
| TPSH5 | Homoeriodictyol | CYP1B1 |
| TPSH6 | Isosakuranetin(7'-Methylnaringenin) | CYP1B1 |
| TPSH7 | Tamarixetin | CYP1B1 |
| TPSH7 | Tamarixetin | ABCC1 |
| TPSH8 | Di-O-methylquercetin | PLG |
| TPSH9 | Naringenin | CYP19A1 |
| TPSH9 | Naringenin | ABCC1 |
| TPSH9 | Naringenin | HSD17B1 |
| TPSH9 | Naringenin | SHBG |
| TPSH9 | Naringenin | CYP1B1 |
| TPSH9 | Naringenin | ESR1 |
| TPSH9 | Naringenin | ESR2 |
| TPSH10 | Naringenin chalcone | EGFR |
| TPSH13 | Apigenin | FLT3 |
| TPSH13 | Apigenin | CYP19A1 |
| TPSH13 | Apigenin | ESR1 |
| TPSH13 | Apigenin | CDK1 |
| TPSH13 | Apigenin | PTGS2 |
| TPSH13 | Apigenin | ESR2 |
| TPSH13 | Apigenin | CDK6 |
| TPSH13 | Apigenin | GSK3B |
| TPSH13 | Apigenin | ABCC1 |
| TPSH13 | Apigenin | HSD17B1 |
| TPSH13 | Apigenin | TTR |
| TPSH13 | Apigenin | CFTR |
| TPSH13 | Apigenin | CYP1B1 |
| TPSH13 | Apigenin | ABCG2 |
| TPSH13 | Apigenin | ALOX5 |
| TPSH13 | Apigenin | PARP1 |
| TPSH13 | Apigenin | ABCB1 |
| TPSH14 | Scopoletin(7-Hydroxy-5-methoxycoumarin) | CA9 |
| TPSH15 | 13-Hydroxy-9, 11-octadecadienoic acid | PPARG |
| TPSH16 | 9-Hydroxy-10, 12-octadecadienoic acid | PPARG |
| TPSH17 | Myristic Acid | PPARD |
| TPSH18 | Pentadecanoic Acid | PPARD |
| TPSH19 | Palmitoleic Acid | PPARG |
| TPSH19 | Palmitoleic Acid | PPARD |
| TPSH20 | γ-Linolenic Acid | PPARG |
| TPSH20 | γ-Linolenic Acid | PPARD |
| TPSH20 | γ-Linolenic Acid | PTGS1 |
| TPSH21 | α-Linolenic Acid | PTGS1 |
| TPSH21 | α-Linolenic Acid | PPARG |
| TPSH21 | α-Linolenic Acid | PPARD |
| TPSH22 | Cis-10-Heptadecenoic Acid | PPARG |
| TPSH22 | Cis-10-Heptadecenoic Acid | TERT |
| TPSH22 | Cis-10-Heptadecenoic Acid | PPARD |
| TPSH28 | L-Glutamic acid | ADORA3 |
| TPSH29 | (-)-3-(3,4-Dihydroxyphenyl)-2-methylalanine | EGFR |
| TPSH31 | 5-Hydroxy-L-tryptophan | HTR2A |
| TPSH33 | 3, 4-Dihydroxy-DL-phenylalanine | EGFR |
| TPSH33 | 3, 4-Dihydroxy-DL-phenylalanine | LCK |
| TPSH35 | Spermine | CASP2 |
| TPSH37 | Ferulic acid | CA9 |
| TPSH38 | Syringic acid | CA9 |
| TPSH40 | Trans-4-Hydroxycinnamic Acid Methyl Ester | CA9 |
| TPSH41 | Trans-ferulic acid | CA9 |
| TPSH42 | Caffeic acid | ALOX5 |
| TPSH42 | Caffeic acid | MMP9 |
| TPSH42 | Caffeic acid | MMP1 |
| TPSH42 | Caffeic acid | MMP2 |
| TPSH42 | Caffeic acid | PTPN1 |
| TPSH42 | Caffeic acid | CA9 |
| TPSH45 | p-Coumaric acid | ESR2 |
| TPSH45 | p-Coumaric acid | CA9 |
| TPSH46 | Gentisic acid | CA9 |
| TPSH47 | Hexadecanoic acid | PPARD |
| TPSH55 | Benzamidine | F2 |
| TPSH56 | Rutundic acid | PTPN1 |
| TPSH57 | p-hydroxy cinnamic acid | ESR2 |
| TPSH57 | p-hydroxy cinnamic acid | CA9 |
| TPSH58 | 3', 5, 5', 7 -tetrahydroxyflavanone | CYP19A1 |
| TPSH58 | 3', 5, 5', 7 -tetrahydroxyflavanone | CYP1B1 |

**Table S4. KEGG pathway enrichment**

| **GO** | **Description** | **LogP** | **Count** | **Hits** |
| --- | --- | --- | --- | --- |
| hsa05200 | Pathways in cancer | -9.984141 | 10 | CDK6\|EGFR\|FLT3\|GSK3B\|MMP1\|MMP2\|MMP9\|PPARD\|PPARG\|PTGS2 |
| hsa05206 | MicroRNAs in cancer | -6.800936 | 7 | CDK6\|CYP1B1\|EGFR\|MMP9\|ABCC1\|ABCB1\|PTGS2 |
| hsa04913 | Ovarian steroidogenesis | -8.103524 | 5 | ALOX5\|CYP1B1\|CYP19A1\|HSD17B1\|PTGS2 |
| hsa01522 | Endocrine resistance | -6.663229 | 5 | EGFR\|ESR1\|ESR2\|MMP2\|MMP9 |
| hsa04915 | Estrogen signaling pathway | -6.618365 | 5 | EGFR\|ESR1\|ESR2\|MMP2\|MMP9 |
| hsa05224 | Breast cancer | -5.787993 | 5 | CDK6\|EGFR\|ESR1\|ESR2\|GSK3B |
| hsa04657 | IL-17 signaling pathway | -5.073336 | 4 | GSK3B\|MMP1\|MMP9\|PTGS2 |
| hsa05219 | Bladder cancer | -6.510951 | 4 | EGFR\|MMP1\|MMP2\|MMP9 |
| hsa05205 | Proteoglycans in cancer | -3.748848 | 4 | EGFR\|ESR1\|MMP2\|MMP9 |
| hsa02010 | ABC transporters | -6.384973 | 4 | CFTR\|ABCC1\|ABCB1\|ABCG2 |
| hsa04726 | Serotonergic synapse | -4.738433 | 4 | ALOX5\|HTR2A\|PTGS1\|PTGS2 |
| hsa04080 | Neuroactive ligand-receptor interaction | -3.23706 | 4 | ADORA3\|F2\|HTR2A\|PLG |
| hsa00140 | Steroid hormone biosynthesis | -4.138656 | 3 | CYP1B1\|CYP19A1\|HSD17B1 |
| hsa04976 | Bile secretion | -3.876321 | 3 | CFTR\|ABCB1\|ABCG2 |
| hsa04917 | Prolactin signaling pathway | -3.894657 | 3 | ESR1\|ESR2\|GSK3B |
| hsa04110 | Cell cycle | -3.16375 | 3 | CDK1\|CDK6\|GSK3B |
| hsa00590 | Arachidonic acid metabolism | -4.05193 | 3 | ALOX5\|PTGS1\|PTGS2 |
| hsa03320 | PPAR signaling pathway | -3.858252 | 3 | MMP1\|PPARD\|PPARG |
| hsa04540 | Gap junction | -3.600058 | 3 | CDK1\|EGFR\|HTR2A |
| hsa04064 | NF-kappa B signaling pathway | -3.502118 | 3 | PARP1\|LCK\|PTGS2 |
| hsa05202 | Transcriptional misregulation in cancer | -2.697998 | 3 | FLT3\|MMP9\|PPARG |
| hsa05166 | HTLV-I infection | -2.268218 | 3 | GSK3B\|LCK\|TERT |

**Table S5. The top ten important components**

| Type | Name | CAS | Degree | Betweenness centrality | Closeness centrality |
| --- | --- | --- | --- | --- | --- |
| TPSH13 | Apigenin | 520-36-5 | 17 | 0.63593496 | 0.46067416 |
| TPSH9 | Naringenin | 480-41-1 | 7 | 0.38109756 | 0.33884298 |
| TPSH42 | Caffeic acid | 331-39-5 | 6 | 0.54292929 | 0.38317757 |
| TPSH20 | γ-Linolenic Acid | 506-26-3 | 3 | 0.19353659 | 0.54545455 |
| TPSH21 | α-Linolenic Acid | 463-40-1 | 3 | 0.12617886 | 0.54545455 |
| TPSH22 | Cis-10-Heptadecenoic Acid | 29743-97-3 | 3 | 0.30487805 | 0.54545455 |
| TPSH7 | Tamarixetin | 603-61-2 | 2 | 0.21212121 | 0.26797386 |
| TPSH19 | Palmitoleic Acid | 373-49-9 | 2 | 0.12121212 | 0.5 |
| TPSH33 | 3,4-Dihydroxy-DL-phenylalanine | 63-84-3 | 2 | 0.25853659 | 0.66666667 |
| TPSH45 | p-Coumaric acid | 501-98-4 | 2 | 0.83333333 | 0.36283186 |

**Table S6. The top ten important targets**

| Name | Degree | Betweenness centrality | Closeness centrality |
| --- | --- | --- | --- |
| ESR1 | 21 | 0.18475896 | 0.72727273 |
| EGFR | 20 | 0.19783796 | 0.72727273 |
| PTGS2 | 19 | 0.19162732 | 0.69565217 |
| MMP9 | 12 | 0.04575647 | 0.60377358 |
| MMP2 | 11 | 0.0743185 | 0.58181818 |
| PPARG | 11 | 0.03602791 | 0.57142857 |
| CYP1B1 | 9 | 0.01561275 | 0.55172414 |
| ESR2 | 9 | 0.01375646 | 0.54237288 |
| PLG | 9 | 0.0498891 | 0.57142857 |
| ABCG2 | 8 | 0.01979647 | 0.52459016 |
| CYP19A1 | 8 | 0.00959372 | 0.53333333 |

**Table S7. The binding energy values of the core active components and Target Proteins**

| **Targets**  **Indigents** | **ESR1** | **EGFR** | **PTGS2** | **MMP9** | **MMP2** | **PPARG** |
| --- | --- | --- | --- | --- | --- | --- |
| **Apigenin** | -4.35 | -4.29 | **-6.31** | **-5.20** | -3.48 | -4.11 |
| **Naringenin** | -4.14 | -3.92 | **-6.20** | **-5.81** | -3.15 | -4.20 |
| **Caffeic acid** | -4.68 | -3.28 | -3.87 | -3.17 | -1.68 | -3.84 |
| **γ-Linolenic Acid** | -3.13 | -2.10 | -2.66 | -1.43 | -0.13 | -2.47 |
| **α-Linolenic Acid** | -2.87 | -1.15 | -3.12 | -1.24 | -0.29 | -2.20 |
| **Cis-10-Heptadecenoic Acid** | -3.89 | -1.53 | -2.99 | -1.34 | -0.12 | -1.88 |


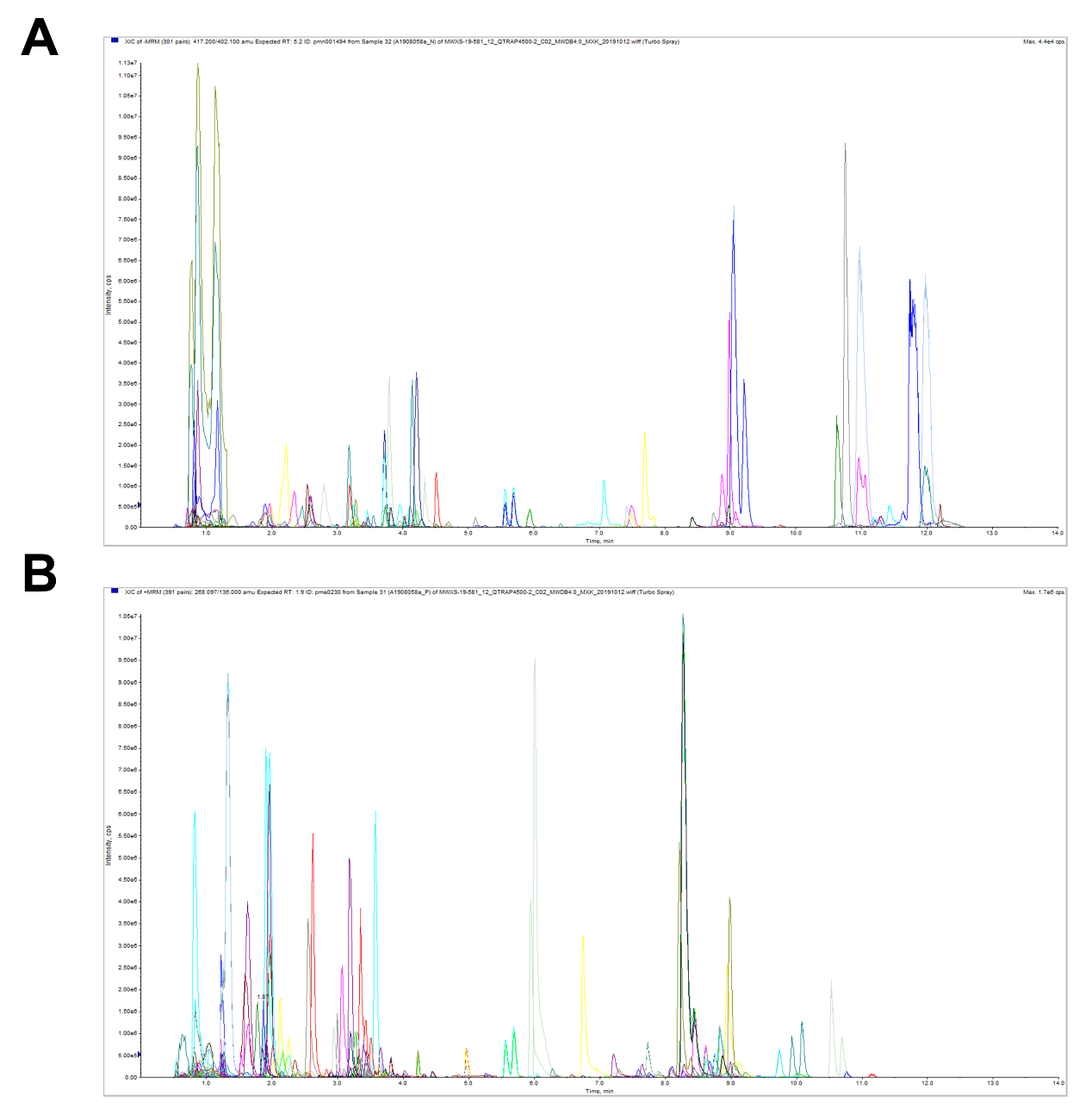


Figure S1. Total ion flow chart of 70% aqueous methanol of *D. officinale*. (A) ESI (–); (B) ESI (+).


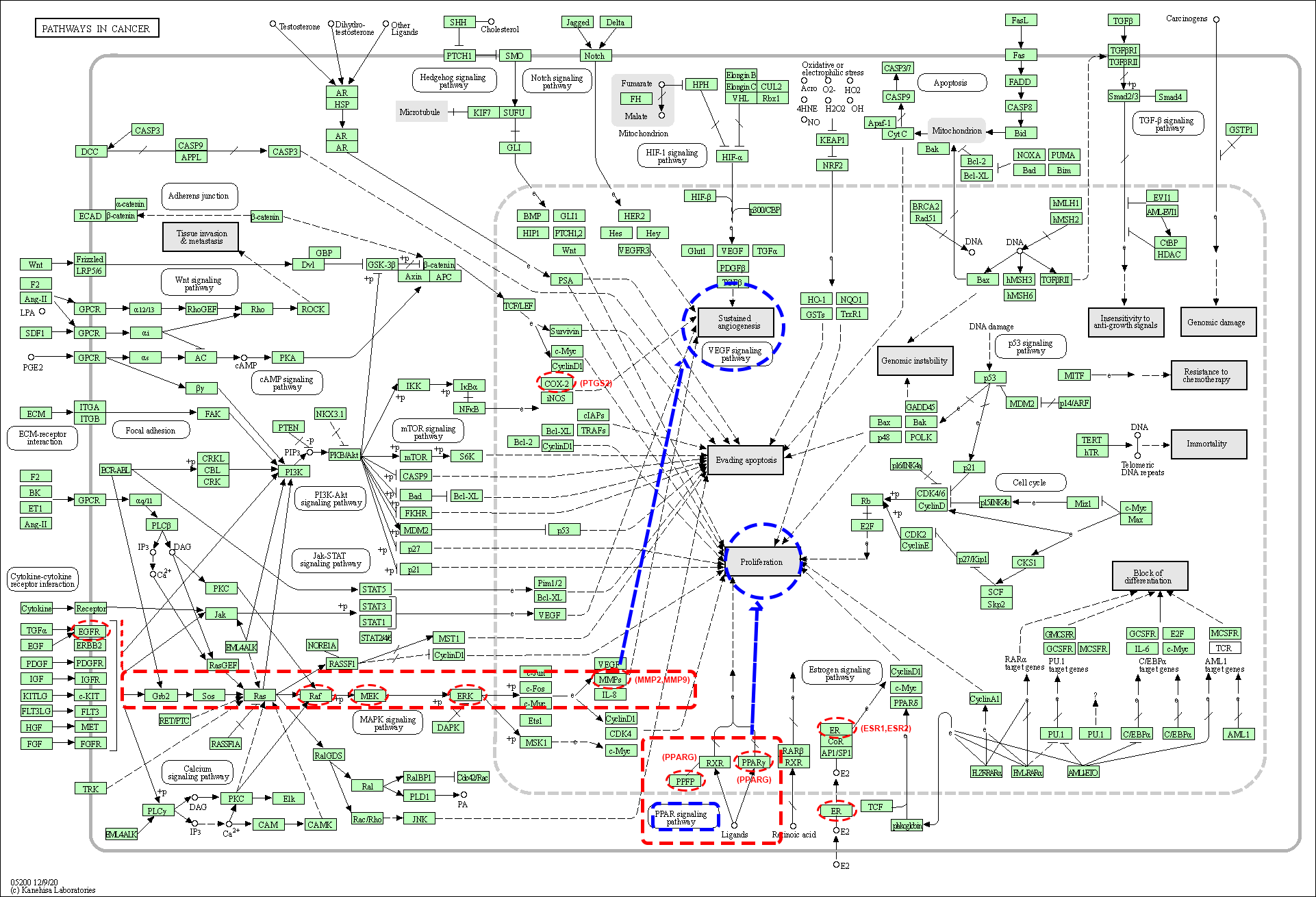


Figure S2. pathways in cancer (https://www.kegg.jp/pathway/hsa05200)


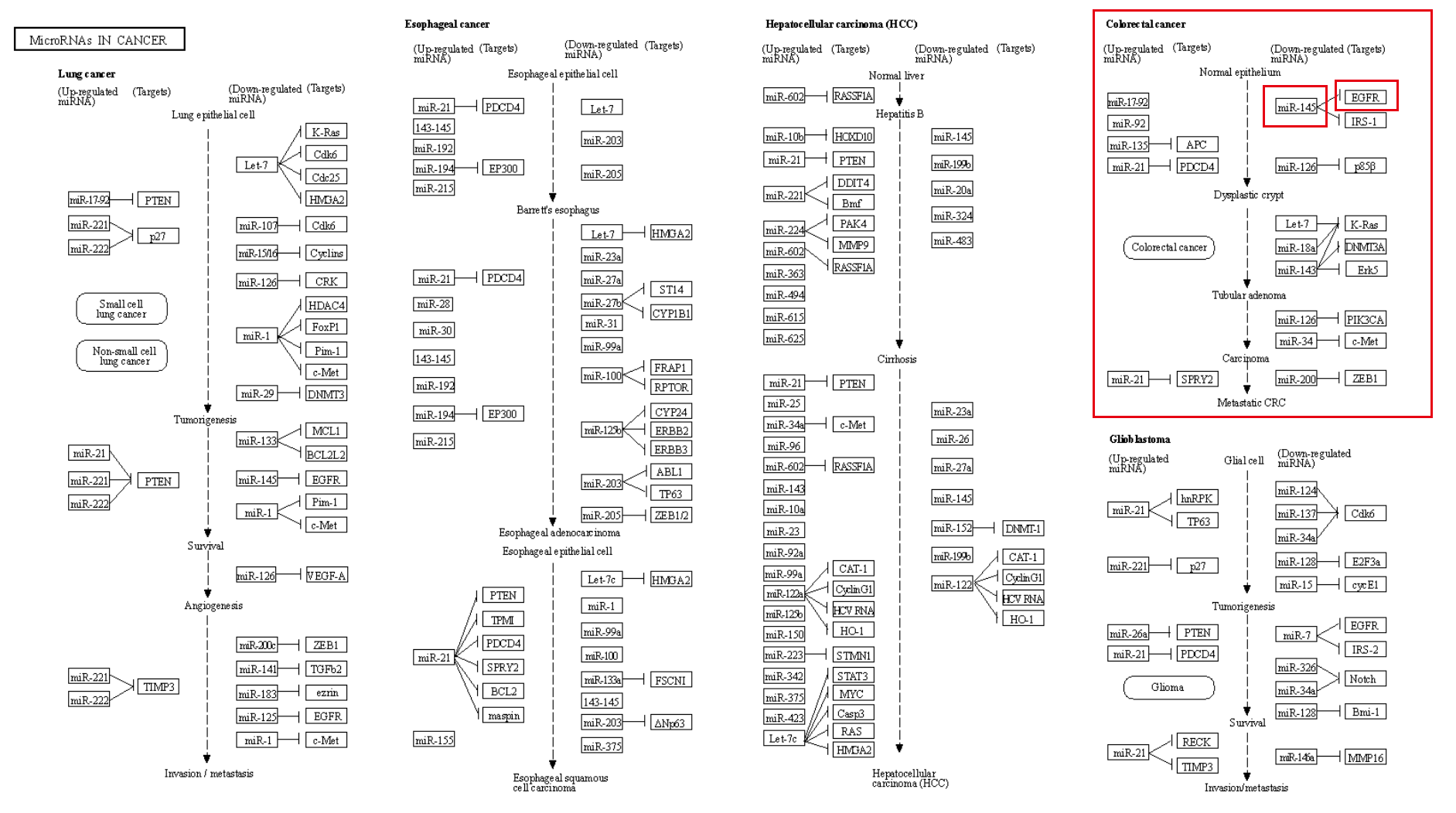


Figure S3. MirRNA in Cancer (https://www.kegg.jp/pathway/map05206)


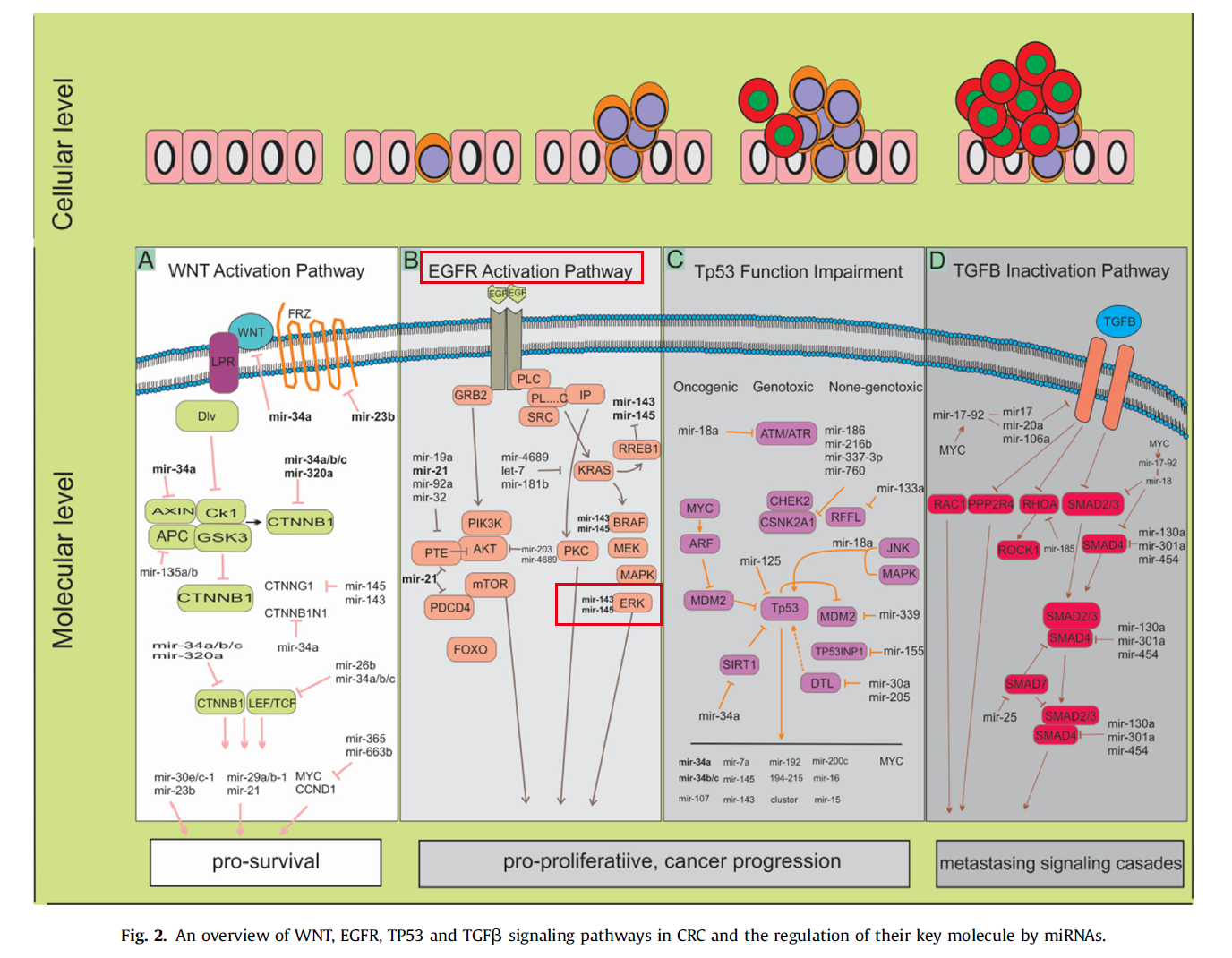


Figure S4. An overview of WNT, EGFR, TP53 and TGFb signaling pathways in CRC and the regulation of their key molecule by miRNAs.[14]

**References**

1 Li Y, Wang CL, Guo SX, Yang JS, Xiao PG. Two new compounds from dendrobium candidum. Chem Pharm Bull 2008;56:1477—1479.

2 Li Y, Wang CL, Wang YJ, Guo SX, Yang JS, Chen XM, Xiao PG. Three new bibenzyl derivatives from dendrobium candidum. Chem Pharm Bull 2009;57:218—219

3 Li Y, Wang CL, Wang YJ, Wang FF, Guo SX, Yang JS, Xiao PG. Four new bibenzyl derivatives from dendrobium candidum. Chem Pharm Bull 2009;57:997—999.

4 Li Y. Studies on the chemical constituents of *dendrobium candidum*. Beijing: Chinese Academy of Medical Sciences & Peking Union Medical College, 2009.

5 Sun J. Study on the inhibitory effects of *dendrobium candidum* extract and its active ingredient erianin on human breast cancer cells. Jilin: Jilin University, 2016.

6 Li Y, Wang CL, Wang FF, Dong Hl, Guo SX, Yang JS, Xiao PG. Chemical constituents of *dendrobium candidum*. China Journal of Chinese Meteria Medica 2010;35:1715-1719.

7 Guan HJ, Zhang X, Tu FJ, Yao XS. Study on chemical constituents of *dendrobium candidum*. Chinese Traditional and Herbal Drugs 2009;40:1873-1876.

8 Li RS, Yang X, He P, Gan N. Studies on phenanthrene constituents from stems of *dendrobium candidum*. Journal of Chinese Medicinal Materials 2009;32:220-223.

9 Li L, Deng XL, Zhao XB, Zeng Y, Ouyang DS. Advances in studies on chemical constituents in *dendrobium candidum* and their pharmacological effects. Anti-tumor Pharmacy 2011;01:90-94.

10 Cui YD, Lu YL, Zhao YM, Liu MX, Zhang GG. Isolation and identification of chemical constituents from *dendrobium officinale* kimura et migo. Journal of Shenyang Pharmaceutical University 2019;36:7-11.

11 Liu MX, Cui YD, Deng BW, Shi S, Zhang CY, Zhang GG. Isolation and identification of chemical constituents from *dendrobium officinale* kimura et migo. Journal of Shenyang Pharmaceutical University 2018;35:739-743+749.

12 Li Y, Wang CL, Wang FF, Dong HL, Guo SX, Yang JS, Xiao PG. Phenolic components and flavanones from *dendrobium candidum*. Chinese Pharmaceutical Journal 2010;45:975-979.

13 Wang FF, Li Y, Dong HL, Guo SX, Wang CL, Yang JS. A new compound from *dendrobium candidum*. Chinese Pharmaceutical Journal 2010;45:898-902.

14 Mohammadi A, Mansoori B, Baradaran B. The role of micrornas in colorectal cancer. Biomed Pharmacother 2016;84:705-713.
